# GBPs Inhibit Motility of *Shigella flexneri* but Are Targeted for Degradation by the Bacterial Ubiquitin Ligase IpaH9.8

**DOI:** 10.1016/j.chom.2017.09.007

**Published:** 2017-10-11

**Authors:** Michal P. Wandel, Claudio Pathe, Emma I. Werner, Cara J. Ellison, Keith B. Boyle, Alexander von der Malsburg, John Rohde, Felix Randow

**Affiliations:** 1 MRC Laboratory of Molecular Biology, Division of Protein and Nucleic Acid Chemistry, Francis Crick Avenue, Cambridge CB2 0QH, UK; 2 Department of Microbiology and Immunology, Dalhousie University Halifax, Halifax, NS B3H 4R2, Canada; 3 University of Cambridge, Department of Medicine, Addenbrooke’s Hospital, Cambridge CB2 0QQ, UK

**Keywords:** cell-autonomous immunity, innate immunity, host-pathogen interaction, effector proteins, E3 ubiquitin ligase, Ipa9.8, ubiquitin, ubiquitin coat, GBP

## Abstract

Interferon exposure boosts cell-autonomous immunity for more efficient pathogen control. But how interferon-enhanced immunity protects the cytosol against bacteria and how professionally cytosol-dwelling bacteria avoid clearance are insufficiently understood. Here we demonstrate that the interferon-induced GTPase family of guanylate-binding proteins (GBPs) coats *Shigella flexneri* in a hierarchical manner reliant on GBP1. GBPs inhibit actin-dependent motility and cell-to-cell spread of bacteria but are antagonized by IpaH9.8, a bacterial ubiquitin ligase secreted into the host cytosol. IpaH9.8 ubiquitylates GBP1, GBP2, and GBP4 to cause the proteasome-dependent destruction of existing GBP coats. This ubiquitin coating of *Shigella* favors the pathogen as it liberates bacteria from GBP encapsulation to resume actin-mediated motility and cell-to-cell spread. We conclude that an important function of GBP recruitment to *S. flexneri* is to prevent the spread of infection to neighboring cells while IpaH9.8 helps bacterial propagation by counteracting GBP-dependent cell-autonomous immunity.

## Highlights

• Guanylate-binding proteins (GBPs) coat *S. flexneri* in a GBP1-dependent manner
• GBP coats antagonize bacterial actin-dependent motility and cell-to-cell spreading
• IpaH9.8, a bacterial E3 ubiquitin ligase, targets GBPs for degradation
• IpaH9.8 re-establishes bacterial motility and cell-to-cell spread

## Graphical Abstract

**Figure:**
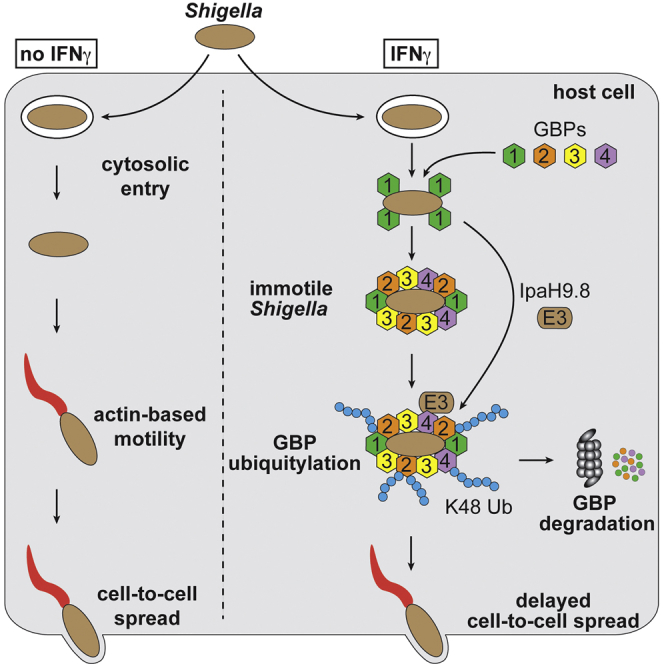

## Teaser

How interferon-enhanced immunity protects cells against bacteria and how cytosol-dwelling bacteria, such as *Shigella*, avoid clearance remain poorly understood. Wandel, Pathe et al. demonstrate that interferon-induced guanylate-binding proteins (GBPs) coat *Shigella* in a GBP1-dependent manner and inhibit actin-dependent cell-to-cell spread of bacteria unless degraded through the bacterial ubiquitin ligase IpaH9.8.

## Introduction

Pathogens inhabit specific niches in their host organism to which they are exquisitely adapted. The host cytosol appears a particularly hostile environment, considering the small number of bacteria able to replicate in this compartment despite its high nutrient content. In resting cells, anti-bacterial autophagy, inflammasome activation, and the induction of cell death are all potent effector mechanisms against cytosol-invading bacteria (Randow et al., 2013). Additional resistance to infection is caused by exposure to interferons, which induce a large number of interferon-stimulated genes (ISGs) to enhance cytosolic immunity (MacMicking, 2012). ISGs antagonize both viruses and bacteria, although their contribution to the interferon-induced “anti-viral” state is much better understood than their anti-bacterial action (Boxx and Cheng, 2016).

Among the interferon-induced effector proteins with anti-bacterial function are guanylate-binding proteins (GBPs), which belong to a large IFN-induced GTPase family (Kim et al., 2012, Man et al., 2017). GBPs have been suggested to compromise the structural integrity of bacteria, to release ligands that stimulate inflammasomes, and to activate anti-bacterial effector mechanisms such as xenophagy and the oxidative burst (Kim et al., 2011, Man et al., 2016, Meunier et al., 2015). The target of GBP action remains hotly disputed since evidence has emerged for GBPs attacking host membranes as well as bacterial surfaces (Man et al., 2016, Meunier et al., 2014). It also remains unknown whether individual GBPs perform specific functions or whether their action is largely redundant as suggested by mouse knockout experiments, in which phenotypes emerged only upon large chromosomal deletions encompassing several GBPs (Yamamoto et al., 2012).

A fundamental question in innate immunity is how professionally cytosol-dwelling bacteria avoid clearance by cell-autonomous immunity (Deretic et al., 2013, Huang and Brumell, 2014). *Shigella flexneri* appears to have evolved sophisticated countermeasures against cytosolic effector mechanisms, such as the IpaH family of E3 ubiquitin ligases, characterized by an N-terminal leucine-rich repeat domain and a uniquely folded C-terminal catalytic domain distinct from cellular E3 ligases (Huibregtse and Rohde, 2014, Rohde et al., 2007, Singer et al., 2008, Zhu et al., 2008). Secreted via the type 3 secretion system (T3SS), IpaH proteins target host processes critical for anti-bacterial defense. IpaH1.4, for example, degrades the LUBAC subunit HOIP to prevent the activation of NF-κB and the deposition of M1-linked ubiquitin chains on the bacterial surface, even on co-infecting *Salmonella enterica* serovar Typhimurium (*S.* Typhimurium) (de Jong et al., 2016, Noad et al., 2017), while IpaH9.8 degrades the IKK subunit NEMO and the splicing factor U2AF35, resulting in reduced inflammation and higher bacterial counts (Ashida et al., 2010, Okuda et al., 2005). On the other hand, cytosol-dwelling bacteria take advantage of the unique opportunities their cytosolic lifestyle offers and infect neighboring cells via actin-dependent motility, thus avoiding exposure to the hostile extracellular space, while host cells carefully monitor their cytosolic homeostasis and deploy septins to antagonize such actin-driven bacterial motility (Mostowy et al., 2010). As demonstrated in primate models of infection, cell-to-cell spread is indeed an essential virulence determinant for *S. flexneri* and inactivating *icsA*, the gene that mediates actin-based motility, may contribute toward development of a live-attenuated vaccine strain (Bernardini et al., 1989, Sansonetti and Arondel, 1989).

By studying the fate of *S. flexneri* in human cells activated with IFNγ, we discovered that, in a hierarchical manner and dependent on GBP1, bacteria become coated with multiple GBPs. The GBP coat antagonizes actin-dependent bacterial motility and invasion of neighboring cells. However, efficient cell-to-cell spread of *S. flexneri* is restored by IpaH9.8, which, by ubiquitylating GBPs for proteasome-dependent degradation, removes existing GBP coats and induces a transient ubiquitin coat. The IpaH9.8-derived ubiquitin coat appears morphologically indistinguishable but functionally opposite to the classical host-generated ubiquitin coat on cytosol-invading bacteria in that it promotes the pathogen rather than immunity.

## Results

### IFNγ Induces Coating of *S. flexneri* with K48-Linked Ubiquitin Chains

**Figure 1:**
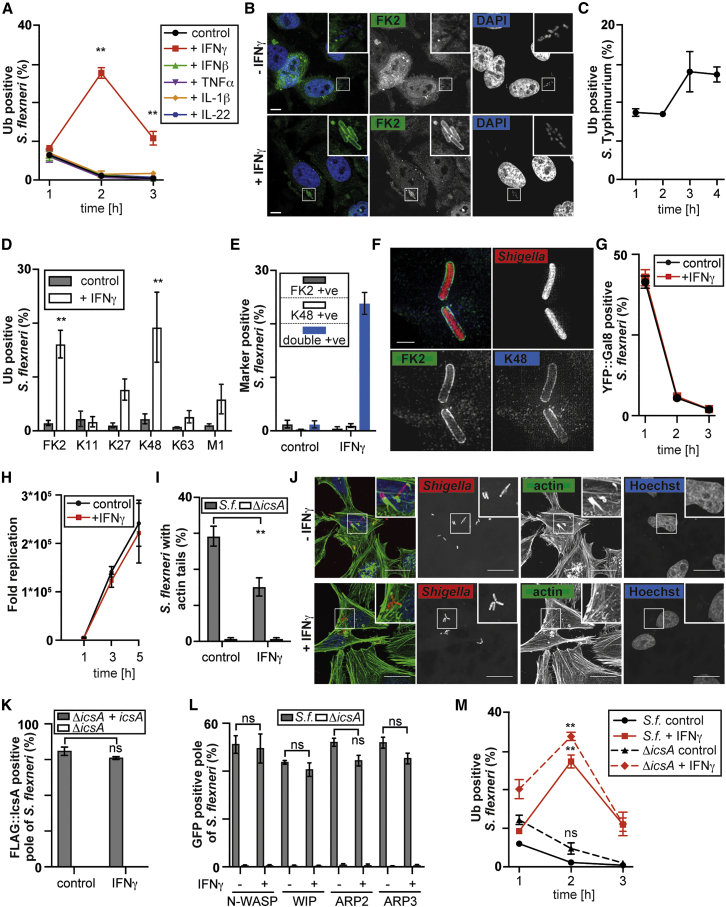
The Effect of IFNγ on Ubiquitin Coating of *S. flexneri* and Actin Tail Formation (A and M) Percentage of *S. flexneri* positive for total ubiquitin in HeLa cells treated with the indicated cytokines. Mean ± SEM of triplicate coverslips from three independent repeats, n > 100 (for 1 hr p.i.), n > 200 (for 2 hr p.i.), n > 300 (for 3 hr p.i.) bacteria per coverslip. ns, non-significant; ^∗∗^p < 0.01, one-way ANOVA with Tukey’s multiple comparisons test. (B and J) Confocal micrographs of HeLa cells treated or untreated with IFNγ and infected with (B) WT or (J) Ruby-expressing *S. flexneri* taken at 2 hr p.i. and stained for (B) total ubiquitin or (J) actin. Scale bar, 10 (B) or 25 μm (J). (C) Percentage of *S.* Typhimurium positive for total ubiquitin in HeLa cells. Mean ± SEM of triplicate coverslips from three independent repeats, n > 100 bacteria per coverslip. (D and E) Percentage of *S. flexneri* positive for the indicated ubiquitin chain types in HeLa cells treated or untreated with IFNγ at 2 hr p.i. Mean ± SEM of triplicate coverslips from three independent repeats, n > 200 bacteria per coverslip, ^∗∗^p < 0.01, Student’s t test. (F) Structured illumination (SI) micrographs of HeLa cells treated with IFNγ and infected with Ruby-expressing *S. flexneri* at 2 hr p.i. and stained for total (FK2) or K48-linked ubiquitin chains. Scale bar, 1 μm. (G) Percentage of *S. flexneri* positive for YFP::galectin-8 in HeLa cells treated or untreated with IFNγ. Mean ± SEM of triplicate coverslips from three independent repeats, n > 100 (for 1 hr p.i.), n > 200 (for 2 hr p.i.), n > 300 (for 3 hr p.i.) bacteria per coverslip. (H) Colony-forming units (CFU) of *S. flexneri* in HeLa cells treated or untreated with IFNγ. Bacteria were counted based on their ability to grow on agar plates. Mean ± SD of triplicate HeLa cultures and duplicate colony counts of a representative experiment. (I) Percentage of WT or Δ*icsA S. flexneri* with actin tails in HeLa cells treated or untreated with IFNγ at 2 hr p.i. Mean ± SEM of triplicate coverslips from three independent repeats, n > 200 bacteria per coverslip, ^∗∗^p < 0.01, Student’s t test. (K) Percentage of Δ*icsA* or Δ*icsA* complemented with FLAG::IcsA *S. flexneri* with FLAG-positive poles in HeLa cells treated or untreated with IFNγ at 2 hr p.i. Mean ± SEM of triplicate coverslips from three independent repeats, n > 100 bacteria per coverslip; ns, non-significant; Student’s t test. (L) Percentage of WT or Δ*icsA S. flexneri* poles positive for the indicated GFP-tagged protein in HeLa cells treated or untreated with IFNγ at 2 hr p.i. Mean ± SEM of triplicate coverslips from three independent repeats, n > 200 bacteria per coverslip; ns, non-significant; Student’s t test.

When entering its replicative niche in the host cytosol, *S. flexneri* avoids becoming coated with polyubiquitin (Figures 1A and 1B), suggesting that lack of ubiquitin coating enables *S. flexneri* to escape xenophagy and other effector mechanisms of cell-autonomous immunity. By contrast, *S.* Typhimurium, which is restricted by autophagy (Birmingham et al., 2006), becomes decorated with polyubiquitin (Figure 1C). To investigate whether exposure of cells to interferons or pro-inflammatory cytokines induces an “anti-bacterial” state, we employed HeLa cells, a well-established model system, to study the entry and subsequent intracellular trafficking of *S. flexneri* in epithelial cells. We found that polyubiquitin accumulated in the vicinity of *S. flexneri* in cells treated with IFNγ, but not IFNβ, TNFα, IL-1β, or IL-22 (Figures 1A and 1B). The ubiquitin coat occurred transiently with a peak at 2 hr post-infection (p.i.) (Figure 1A), consisted mainly of K48-linked ubiquitin chains (Figures 1D and 1E), and was associated with the bacterial surface as revealed by structured illumination microscopy, a super-resolution technique (Figure 1F). Ubiquitin accumulation on *S. flexneri* in IFNγ-treated cells was not caused by enhanced bacterial entry into the host cell cytosol, as indicated by the indistinguishable recruitment of galectin-8 to bacteria (Figure 1G) (Thurston et al., 2012). We conclude that IFNγ modulates a post-entry event, such as, for example, the ability of an E3 ubiquitin ligase to synthesize polyubiquitin in the vicinity of *S. flexneri*. Given the well-established role of the bacterial ubiquitin coat as an “eat-me” signal for anti-bacterial autophagy (Birmingham et al., 2006, von Muhlinen et al., 2013, Perrin et al., 2004, Thurston et al., 2009, Wild et al., 2011), we compared bacterial replication in mock and IFNγ-treated cells. IFNγ did not affect bacterial proliferation (Figure 1H). However, it significantly antagonized the development of actin tails (Figures 1I and 1J), a key feature of *S. flexneri*, induced by IcsA (Bernardini et al., 1989), and strictly required for bacterial motility in the host cytoplasm. However, IFNγ did not affect the typical polar localization of IcsA on bacteria (Figure 1K) or the fraction of bacteria that had attracted N-WASP, WIP, ARP2, or ARP3, all involved in actin tail formation (Figure 1L), suggesting IFNγ acts downstream of their recruitment. We next considered the formal possibility of actin-dependent motility antagonizing the development of the ubiquitin coat. However, *S. flexneri* Δ*icsA*, which completely lacks actin tails (Figure 1I), also became ubiquitin coated only in IFNγ-treated cells, similar to wild-type (WT) bacteria (Figure 1M), indicating that reduced motility of *S. flexneri* does not automatically lead to ubiquitin coating.

### GBP1 Mediates Hierarchical GBP Recruitment and Induces Ubiquitin Coating of *S. flexneri*

**Figure 2:**
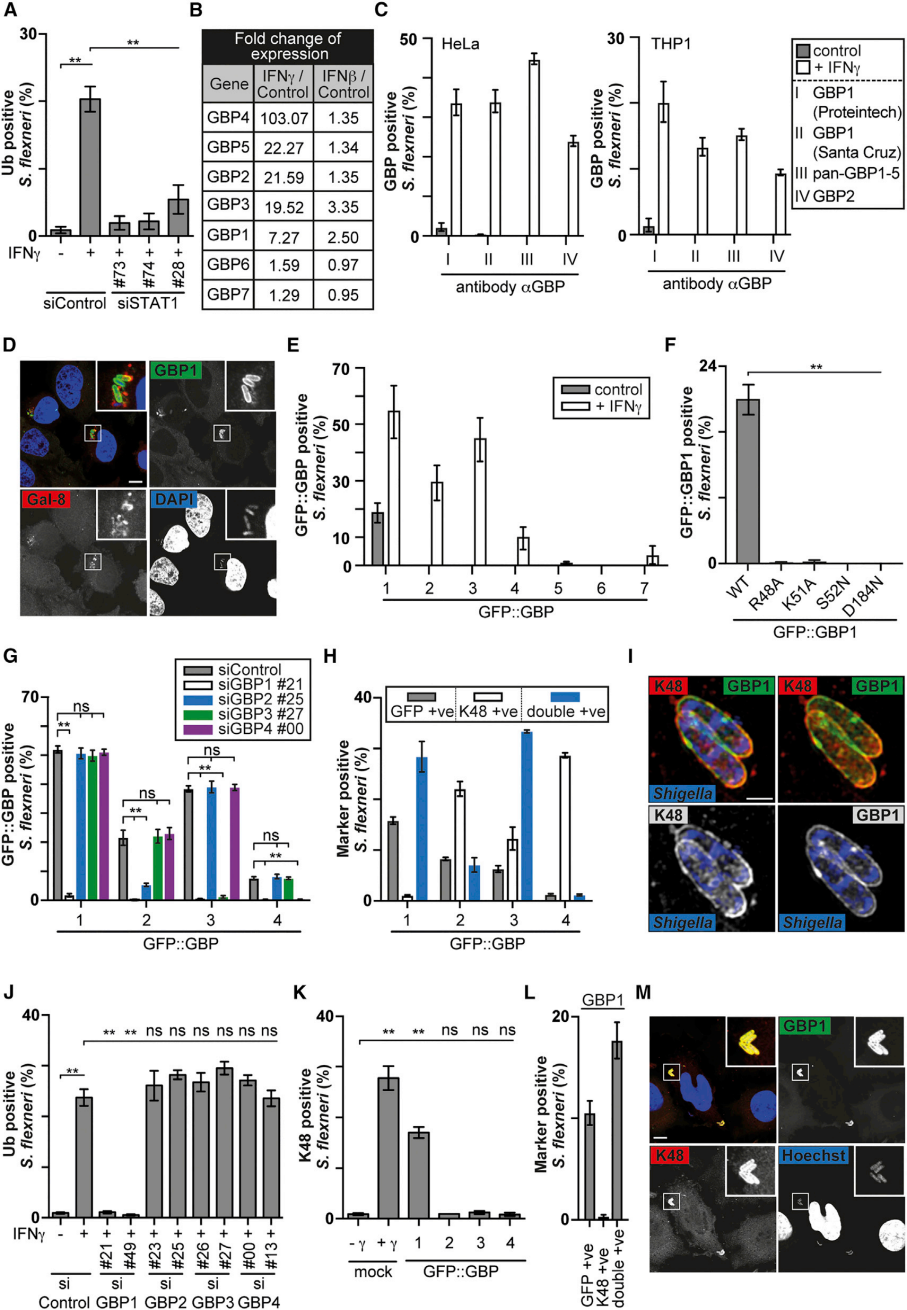
GBP1 Is Required for GBP Recruitment and Ubiquitin Coating of *S. flexneri* (A and J) Percentage of *S. flexneri* positive for total ubiquitin at 2 hr p.i. HeLa cells were treated with siRNAs against (A) STAT1 or (J) GBPs as indicated. Cells were treated or untreated with IFNγ as indicated. Mean ± SEM of triplicate coverslips from three independent repeats, n > 200 bacteria per coverslip. ns, non-significant; ^∗∗^p < 0.01, one-way ANOVA with Tukey’s multiple comparisons test. (B) Microarray analysis of interferon-regulated genes in HeLa cells. Log_2_ fold induction in interferon-treated over untreated cells. (C) Percentage of *S. flexneri* positive for the indicated endogenous GBPs at 1 hr p.i. HeLa cells (left) or THP-1 cells (right) were treated or untreated with IFNγ. Mean ± SEM of triplicate coverslips from three independent repeats, n > 100 bacteria per coverslip. (D and M) Confocal micrographs of (D) HeLa cells treated with IFNγ and stained with endogenous GBP1 antibody (Proteintech) or (M) HeLa cells expressing GFP-tagged GBP1 not treated with IFNγ. Cells were infected with *S. flexneri*, fixed at (D) 1 hr or (M) 2 hr p.i., and stained for (D) galectin-8 or (M) K48-linked ubiquitin. Scale bar, 10 μm. (E and F) Percentage of *S. flexneri* positive for the indicated GFP-tagged GBP alleles at 1 hr p.i. HeLa cells were (F) untreated or (E) treated with INFγ as indicated. Mean ± SEM of triplicate coverslips from three independent repeats, n > 100 bacteria per coverslip. ^∗∗^p < 0.01, one-way ANOVA with Tukey’s multiple comparisons test. (G) Percentage of *S. flexneri* positive for GFP-tagged GBP alleles at 1 hr p.i. HeLa cells were treated with INFγ and with siRNAs against GBPs as indicated. Mean ± SEM of triplicate coverslips from three independent repeats, n > 100 bacteria per coverslip. ns, non-significant; ^∗∗^p < 0.01, one-way ANOVA with Tukey’s multiple comparisons test. (H, K, and L) Percentage of *S.flexneri* positive for (K) K48-linked ubiquitin chains or (H and L) the indicated GFP-tagged GBP alleles and K48-linked ubiquitin chains at 2 hr p.i. HeLa cells were (H) treated, (L) untreated, or (K) treated with INFγ as indicated. Mean ± SEM of triplicate coverslips from three independent repeats, n > 200 bacteria per coverslip. ns, non-significant; ^∗∗^p < 0.01, one-way ANOVA with Tukey’s multiple comparisons test. (I) Structured illumination micrographs of HeLa cells expressing GFP-tagged GBP1 treated with IFNγ and infected with mCherry-expressing *S. flexneri* at 2 hr p.i. and stained for K48-linked ubiquitin. Scale bar, 1 μm. See also Figures S1–S3 and Table S1.

To test whether the induction of the bacterial ubiquitin coat by IFNγ requires *de novo* gene expression, we knocked down STAT1, a transcription factor essential for IFNγ effects. In cells lacking STAT1, *S. flexneri* did not become coated by polyubiquitin (Figures 2A and S1A). To identify the gene(s) responsible for the phenotype, we compared gene expression in cells treated with IFNγ and IFNβ. However, microarray analysis did not reveal any E3 ubiquitin ligase prominently induced by IFNγ (Table S1), suggesting that the IFNγ-dependent coating of *S. flexneri* with polyubiquitin may rather require the induction of gene(s) regulating a pre-existing E3 ligase. GBPs comprise a family of seven GTPases in humans that control cell-autonomous immunity (Man et al., 2017), of which GBP1, GBP2, GBP3, GBP4, and GBP5 were strongly and selectively upregulated by IFNγ (Figure 2B). When investigating the localization of GFP-tagged GBPs in cells stimulated with IFNγ, we observed that GBPs co-localized directly with bacteria and did not stain galectin-8-positive damaged endomembranes in the vicinity of bacteria that had originated from the entry of *S. flexneri* into the cytosol (Figure S2). Antibodies raised against GBP1 or GBP2, or pan-reactive for GBP1–5 revealed that, in both HeLa and THP1 cells, upon stimulation with IFNγ, endogenous GBPs were recruited to *S. flexneri* (Figures 2C and 2D). At 1 hr p.i. between 30% and 60% of *S. flexneri* co-localized with GFP-tagged GBP1, GBP2, and GBP3; 10% with GBP4; and 5% with GBP7, while very few or none were positive for GBP5 and GBP6 (Figure 2E). Overexpressed GBP1 associated with *S. flexneri* even in the absence of IFNγ (Figure 2E). GBP1 recruitment required a catalytically active GTPase domain (Figure 2F), and importantly, GBP1 was essential for the recruitment of GBP2, GBP3, or GBP4 (Figures 2G and S1B). In contrast, depletion of GBP2, GBP3, or GBP4 had no effect on the recruitment of other GBPs. We therefore conclude that in a GTPase-dependent manner, GBP1 performs an essential upstream role in initiating the hierarchical recruitment of GBPs to cytosol-invading bacteria.

We next investigated a potential functional link between GBP recruitment to, and ubiquitin coating of, *S. flexneri*. We found that the majority of GBP1- and GBP3-positive *S. flexneri* were also coated with ubiquitin but that, importantly, only GBP1 occurred on all ubiquitin-coated bacteria (Figures 2H and S3). Structured illumination microscopy revealed an association of both GBP1 and K48-linked ubiquitin chains with the bacterial surface (Figure 2I). Depletion of GBP1 prevented the occurrence of polyubiquitin on *S. flexneri* in cells stimulated with IFNγ (Figure 2J), while overexpression of GBP1 in resting cells was sufficient to induce ubiquitin coating (Figures 2K–2M). Depletion or overexpression of GBP2, GBP3, or GBP4 had no effect. Taken together, we conclude that the ubiquitin coat of *S. flexneri* in cells stimulated with IFNγ is due to the induction of GBP1, which localizes to *S. flexneri*, initiates the hierarchical recruitment of other GBPs, and may recruit an E3 ubiquitin ligase.

### IpaH9.8 Decorates *S. flexneri* with Polyubiquitin

**Figure 3:**
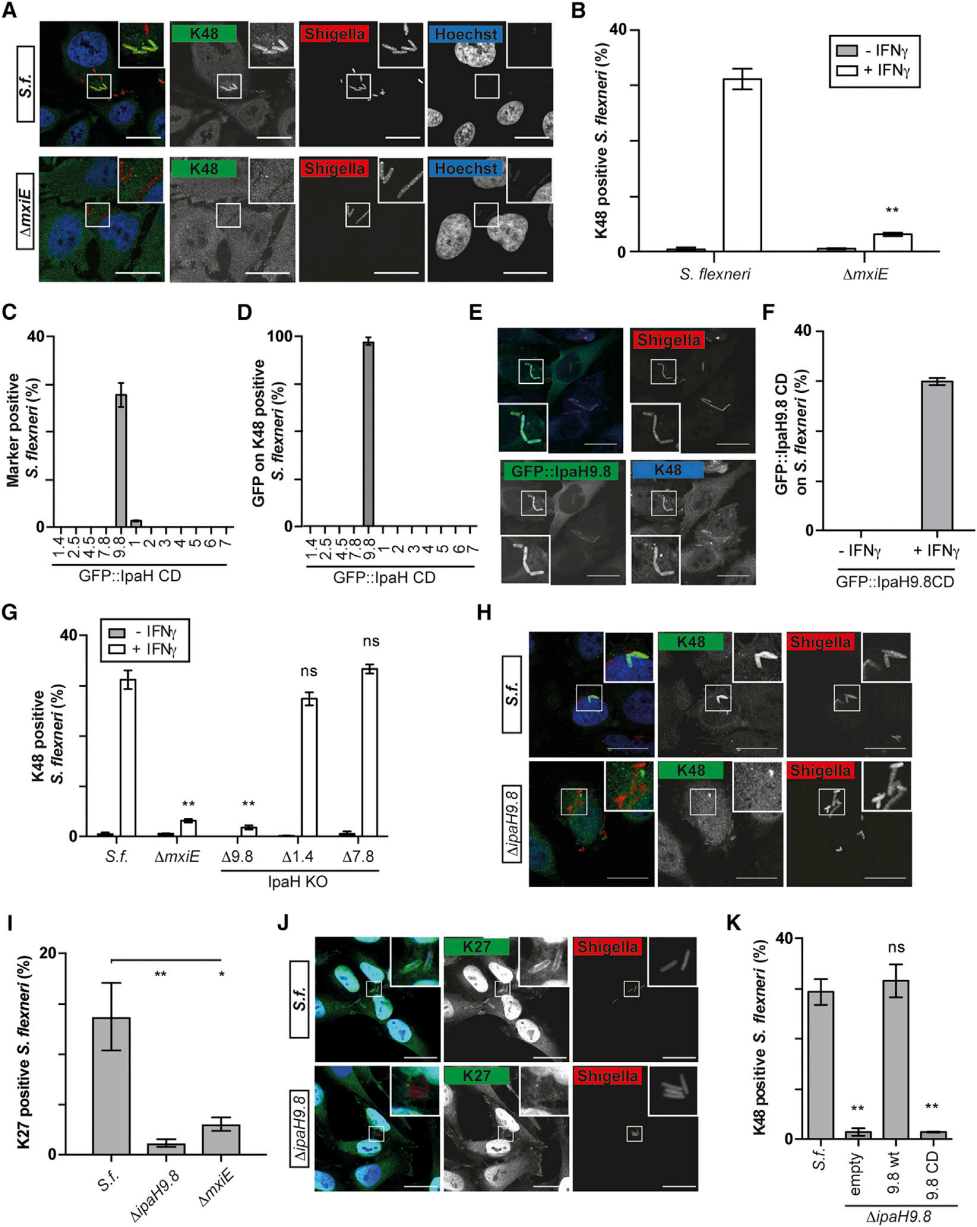
IpaH9.8 Causes Ubiquitin Coats on *S. flexneri* (A, E, H, and J) Confocal micrographs of HeLa cells treated with IFNγ and infected with either WT or the indicated strains of *S. flexneri* expressing Ruby at 2 hr p.i. Cells were stained for (A and H) K48-linked ubiquitin, (J) K27-linked ubiquitin, or (E) K48-linked ubiquitin and expressed GFP-tagged, catalytically inactive IpaH9.8. Scale bar, 25 μm. (B and G) Percentage of the indicated *S. flexneri* strains positive for K48-linked ubiquitin at 2 hr p.i. HeLa cells were treated with IFNγ as indicated. Mean ± SEM of triplicate coverslips from three independent repeats, n > 200 bacteria per coverslip. ns, not significant; ^∗∗^p < 0.01, (B) Student’s t test or (G) one-way ANOVA with Dunnett’s multiple comparisons test. (C, D, and F) HeLa cells expressing inactive alleles of the indicated *ipaH* genes and (C and D) treated with IFNγ or (F) treated with IFNγ as indicated were infected with *S. flexneri*. (C and F) Bacteria positive for the indicated GFP-tagged IpaHs or (D) percentage of bacteria positive for GFP-tagged ipaHs among those positive for K48-linked ubiquitin at 2 hr p.i. Mean ± SEM of triplicate coverslips from three independent repeats, n > 200 bacteria per coverslip. (I and K) Percentage of the indicated *S. flexneri* strains positive for (I) K27-linked ubiquitin or (K) K48-linked ubiquitin in HeLa cells treated with IFNγ at 2 hr p.i. (K) Δ*IpaH9.8* complemented with empty vector, WT, or catalytically inactive alleles of IpaH9.8. Mean ± SEM of triplicate coverslips from three independent experiments, n > 200 bacteria per coverslip. ns, not significant; ^∗^p < 0.05, ^∗∗^p < 0.01, one-way ANOVA with Dunnett’s multiple comparisons test.

We considered the possibility that an E3 ubiquitin ligase encoded by *S. flexneri* rather than a cellular enzyme may generate the bacterial ubiquitin coat. To test our hypothesis, we used *S. flexneri* Δ*mxiE*, a strain deficient in the upregulation of many effector genes upon contact with host cells, including up to twelve bacterially encoded E3 ubiquitin ligases. *S. flexneri* Δ*mxiE* did not become coated with K48-linked ubiquitin chains (Figures 3A and 3B). We therefore investigated whether members of the *Shigella*-encoded and MxiE-controlled IpaH family of E3 ubiquitin ligases are recruited to and mediate ubiquitin coating of *S. flexneri* in IFNγ-stimulated cells. Among a panel of catalytically inactive IpaHs, only IpaH9.8 was recruited to *S. flexneri* (Figure 3C). Recruitment of exogenously expressed, catalytically inactive IpaH9.8 occurred specifically to bacteria that were also coated with K48-linked ubiquitin chains (Figures 3D and 3E) and only in cells stimulated with IFNγ (Figure 3F). To test whether IpaH9.8 is required for ubiquitin coating of *S. flexneri* in IFNγ-stimulated cells, we deployed strains lacking specific *ipaH* genes. *S. flexneri* Δ*ipaH9.8* did not become coated with K48-linked ubiquitin chains, in contrast to *S. flexneri* Δ*ipaH1.4* or Δ*ipaH7.8* (Figures 3G and 3H). An antibody against K27-linked ubiquitin also lost reactivity against *S. flexneri* Δ*ipaH9.8* (Figures 3I and 3J). Complementation with *ipaH9.8*, but not with catalytically inactive *ipaH9.8*_*C337A*_, restored ubiquitin deposition on *S. flexneri* ΔIpaH9.8 (Figure 3K). We therefore conclude that the GBP1-dependent ubiquitin coat of *S. flexneri* in IFNγ-stimulated cells is synthesized by IpaH9.8. The bacterial origin of the ubiquitin coat on *S. flexneri* in cells stimulated with IFNγ suggests that this particular ubiquitin coat may not have anti-bacterial functions but rather that IpaH9.8 counteracts a GBP-dependent cellular defense pathway, potentially through directly antagonizing GBP recruitment.

### IpaH9.8 Ubiquitylates GBPs for Proteasomal Degradation

**Figure 4:**
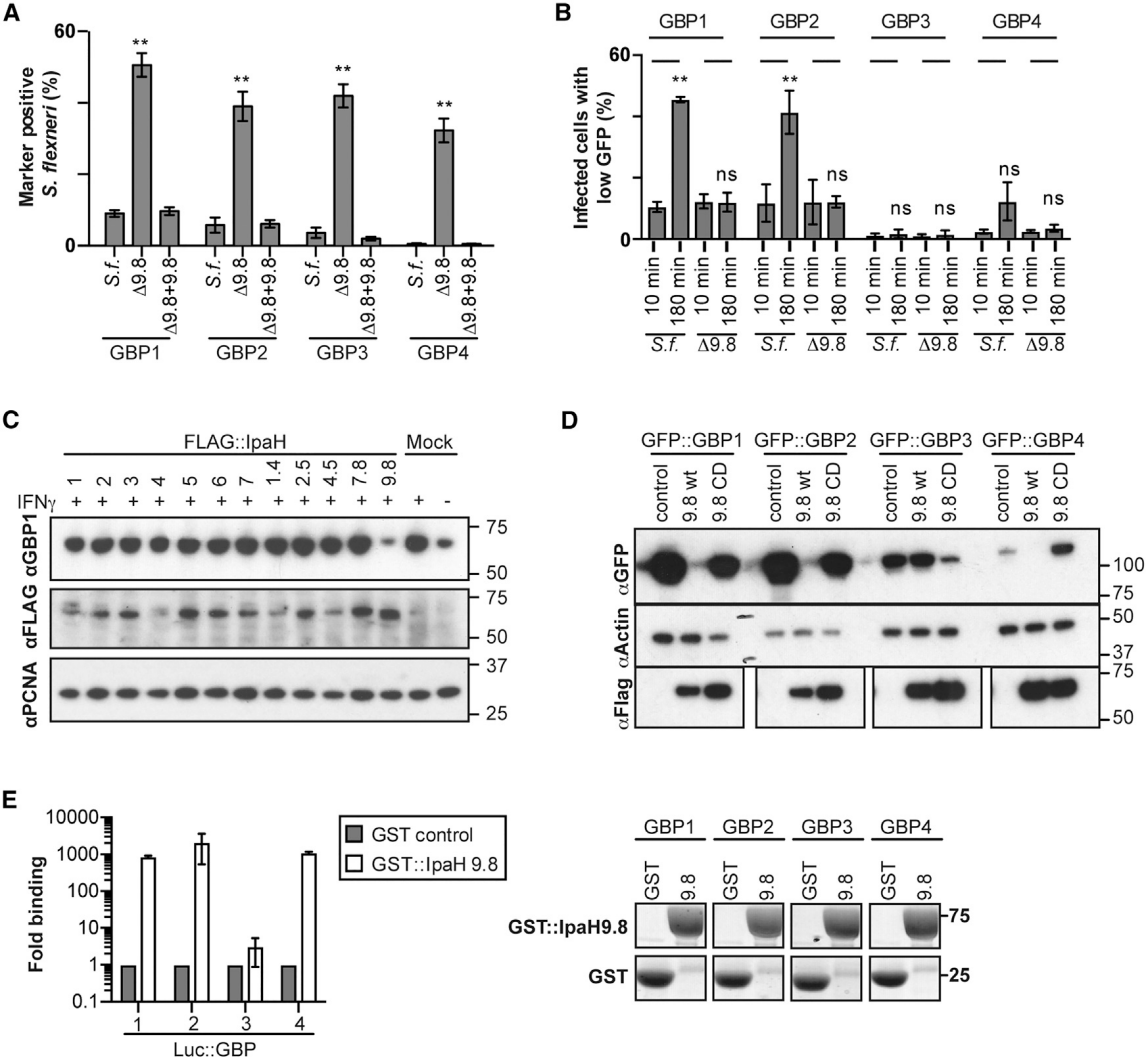
IpaH9.8 Causes GBP Degradation (A) Percentage of the indicated *S. flexneri* strains positive for GFP-tagged GBP1–4 in IFNγ-stimulated HeLa cells at 4 hr p.i. Mean ± SEM of triplicate coverslips from three independent repeats, n > 200 bacteria per coverslip. ^∗∗^p < 0.01, one-way ANOVA with Tukey’s multiple comparisons test (WT against Δ9.8 for each sample). (B) HeLa cells expressing GFP-tagged GBP alleles were analyzed by flow cytometry and gated for intracellular *S. flexneri* expressing Ruby. Infected cells with reduced GBP levels were quantified. Mean ± SD of three independent repeats, n > 10,000 cells per sample. ns, non-significant; ^∗∗^p < 0.01, one-way ANOVA with Tukey’s multiple comparisons test. Dot plots and gating strategy of a representative repeat are shown in Figure S4. (C) Lysates from HeLa cells mock transduced or transduced with the indicated Flag-tagged *ipaH* constructs were treated with IFNγ and probed with antibodies against GBP1, Flag, and PCNA. (D) 293ET cells were co-transfected with GFP-tagged GBP alleles and Flag-tagged *ipaH* constructs as indicated. Lysates were probed for GFP, Flag, and β-actin. (E) LUMIER binding assay. Luciferase-tagged GBP1–4 transfected in 293ET cells were pulled down using recombinant GST or GST::*ipaH9.8* coupled to beads. Proteins were eluted from beads with glutathionine. Left panel: protein binding was determined as luciferase activity over GST control. Mean ± SD of two independent repeats. Right panel: coupling to beads was assessed by SDS-PAGE and Coomassie staining. See also Figure S4.

**Figure 5:**
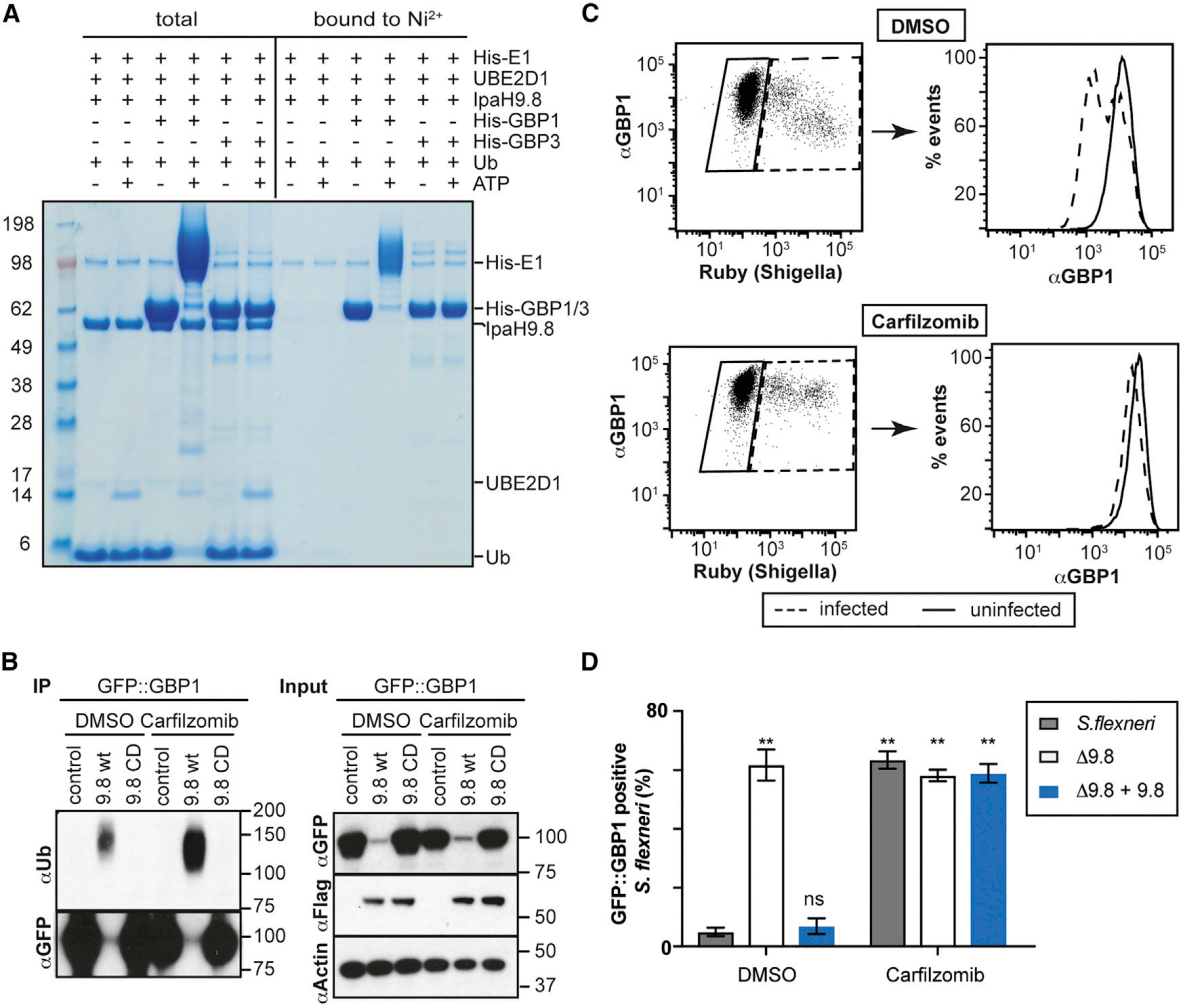
IpaH9.8 Ubiquitylates GBPs for Proteasomal Degradation (A) *In vitro* ubiquitylation of GBPs by IpaH9.8. His_6_-GBP1 or His_6_-GBP3 was incubated with IpaH9.8 in the presence of His_6_-E1, UBE2D1, and ubiquitin in the presence or absence of ATP. Total shows the reaction outcome following incubation for 30 min at 37°C. Ni^2+^ represents His_6_-tagged species from the total reaction after binding to Ni^2+^ resin. (B) 293ET cells were co-transfected with GFP-tagged GBP alleles and Flag-tagged *ipaH* constructs as indicated. Lysates were probed for GFP, Flag, and β-actin. GFP-tagged proteins were immunoprecipitated and probed for GFP and FK2 (IP). (C) Flow cytometry of HeLa cells treated with IFNγ and infected with Ruby-expressing *S. flexneri*. A total of 100 nM Carfilzomib or DMSO was added 30 min p.i. Cells were stained for endogenous GBP1 (Proteintech) and gated for infected (dashed) and uninfected cells (solid). (D) Percentage of the indicated *S. flexneri* strains coated with GFP-tagged GBP1 in IFNγ-stimulated HeLa cells at 4 hr p.i as enumerated by microscopy. A total of 100 nM Carfilzomib was added 30 min p.i. Mean ± SEM of triplicate coverslips from three independent repeats, n > 200 bacteria per coverslip. ns, not significant; ^∗∗^p < 0.01, one-way ANOVA with Dunnett’s multiple comparisons test.

We therefore investigated whether IpaH9.8 negatively controls the accumulation of GBPs on *S. flexneri*. Bacteria lacking IpaH9.8 were much more frequently associated with GBP1, GBP2, GBP3, and GBP4; complementation with IpaH9.8 reversed the effect (Figure 4A). To test whether IpaH9.8 controls GBP accumulation on bacteria by degrading GBPs, we analyzed GBP levels by flow cytometry in cells infected with *S. flexneri*. At 10 min p.i., GBP levels were indistinguishable between infected and uninfected cells, while at 180 min p.i., levels of GBP1, GBP2, and, to a lesser extent, GBP4 were reduced specifically in cells carrying a high burden of *S. flexneri*, i.e., in cells with proliferating bacteria (Figures 4B and S4). GBP3 levels were unaffected. Infection with *S. flexneri* Δ*ipaH9.8* did not reduce GBP levels, revealing that IpaH9.8 is essential for reducing GBP levels in infected cells. Endogenous GBP1 in cells stimulated with IFNγ was similarly reduced upon overexpression of IpaH9.8, but not other IpaH family members (Figure 4C), thus revealing exquisite specificity of IpaH proteins in the absence of other bacterial proteins. To further investigate the specificity of IpaH9.8 and to reveal how GBPs are degraded, we co-expressed IpaH9.8 and GBPs in 293ET cells. Similar to infection with *S. flexneri*, expression of WT, but not catalytically inactive IpaH9.8, drastically reduced levels of GBP1, GBP2, and GBP4, but not GBP3 (Figure 4D). Such specificity of GBP degradation correlates with and is likely explained by the ability of IpaH9.8 to bind GBP1, GBP2, and GBP4, but not GBP3 (Figures 4E and S5). To investigate whether GBPs are direct substrates for IpaH9.8, we performed *in vitro* ubiquitylation assays using purified proteins. IpaH9.8 catalyzed almost quantitative ubiquitylation of GBP1, resulting in a high molecular weight ubiquitin smear, but was inactive toward GBP3 (Figure 5A), consistent with the binding specificity of IpaH9.8 for GBPs (Figure 4E) and the autoinhibited state of IpaH E3 ligases in the absence of bound substrate (Chou et al., 2012). Co-expression of GBP1 and IpaH9.8 resulted in ubiquitylation of GBP1 and its degradation (Figure 5B). Treatment with the proteasome inhibitor Carfilzomib antagonized the effects of IpaH9.8 on GBPs — Carfilzomib caused accumulation of polyubiquitin in immunoprecipitated GBP1 samples (Figure 5B), it rescued the degradation of endogenous GBP1 in cells infected with *S. flexneri* (Figure 5C), and it restored GBP1 coats on *S. flexneri* (Figure 5D). Taken together, we conclude that *S. flexneri* secretes IpaH9.8 to ubiquitylate specific GBPs and cause their proteasome-mediated degradation, resulting in reduced GBP levels in infected cells and escape of bacteria from labeling by GBPs.

### IpaH9.8 Degrades GBPs to Promote Bacterial Spreading

**Figure 6:**
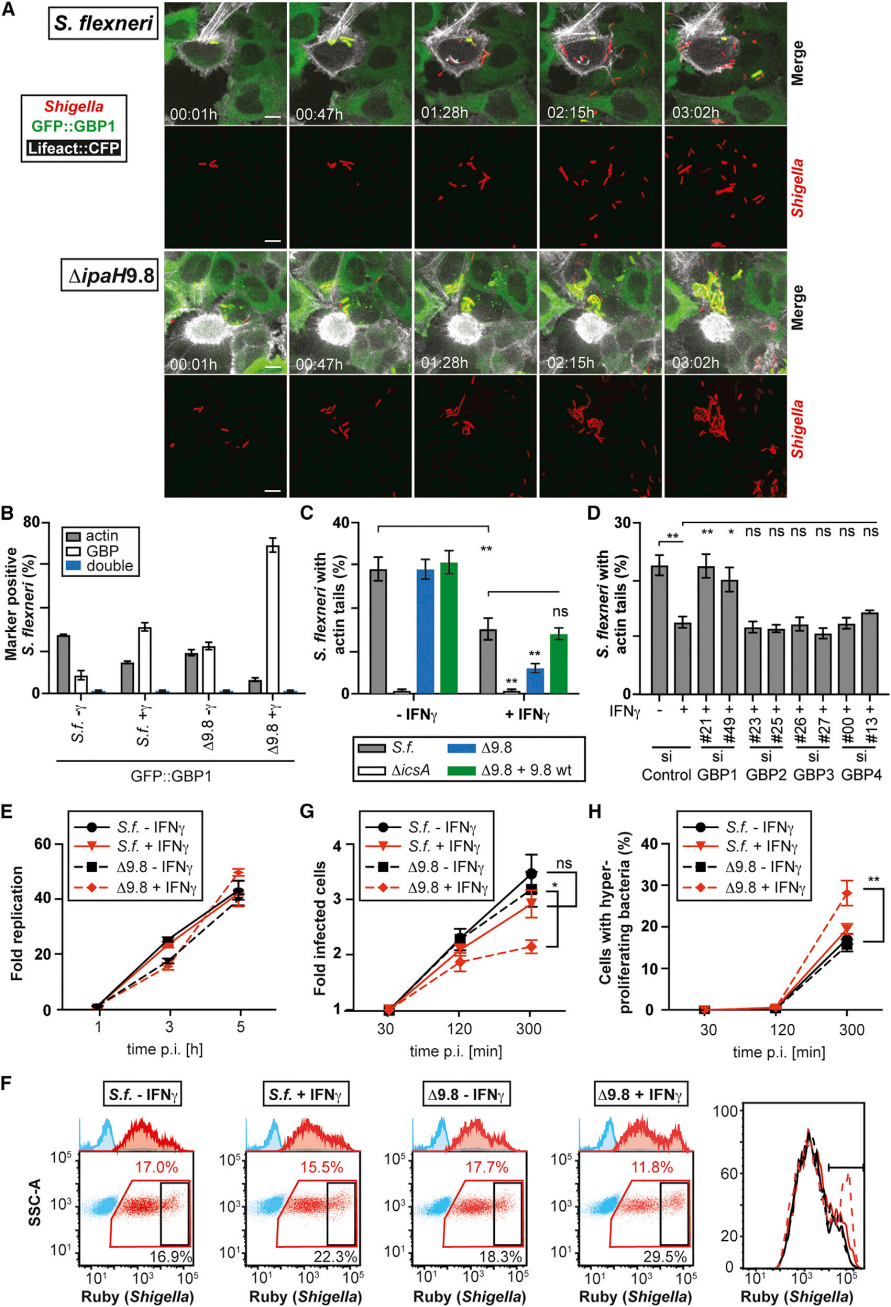
IpaH9.8 Antagonizes GBP Effects on Actin-Dependent Motility and Bacterial Spread (A) Representative frames from Movies S1 and S2 of HeLa cells co-expressing GFP::GBP1 and Lifeact::CFP infected with WT or Δ*ipaH9.8* Ruby-expressing *S. flexneri*. Time p.i. as indicated; scale bar, 10 μm. (B) *S. flexneri* positive for actin tails and/or GBP1 at 2 hr p.i. in HeLa cells expressing GFP::GBP1. Mean ± SEM of triplicate coverslips from three independent repeats, n > 200 bacteria per coverslip. ns, not significant; ^∗∗^p < 0.01 one-way ANOVA with Tukey’s multiple comparisons test. (C and D) *S. flexneri* positive for actin tails at 2 hr p.i. in HeLa cells treated with IFNγ as indicated and (D) treated with indicated siRNAs against human GBPs. Mean ± SEM of triplicate coverslips from three independent repeats, n > 200 bacteria per coverslip. ns, not significant; ^∗^p < 0.05, ^∗∗^p < 0.01, Student’s t test (control) or one-way ANOVA with Tukey’s multiple comparisons test (control versus GBP siRNAs). (E–H) Replication of *S. flexneri* in HeLa cells treated with or without IFNγ and infected with WT or Δ*ipaH9.8 S. flexneri* for the indicated times. (E) Fold replication of *S. flexneri*. Bacteria were counted based on their ability to grow on agar plates. Mean ± SD of triplicate HeLa cultures and duplicate colony counts. (F) Representative fluorescence-activated cell sorting (FACS) dot plots at 300 min p.i. illustrating gating strategy for data in (G) and (H). Uninfected cells are blue; infected cells are red. Infected cells were gated for high levels of Ruby (black). Percentages of events are indicated in corresponding colors. Red, percentage of infected cells; black, percentage of infected cells with high load of *S. flexneri*. Histogram shows infected cells and the gate for cells with high load of *S. flexneri*. (G) Spread of infection by Ruby-expressing *S. flexneri* through HeLa culture scored by flow cytometry as illustrated by red gate in (F). (H) Percentage of infected HeLa cells with high levels of Ruby as illustrated by black gate in (F). (G and H) Mean ± SEM of triplicate HeLa cultures of at least three independent repeats. ^∗^p < 0.05, ^∗∗^p < 0.01, Student’s t test; ns, not significant. See also Figures S5 and S6 and Movies S1 and S2.

To investigate whether the inhibition of actin tail formation on *S. flexneri* observed upon IFNγ treatment is mediated by GBPs and whether GBP degradation by IpaH9.8 precedes actin-driven motility of *S. flexneri*, we performed live microscopy in cells expressing GFP::GBP1 and labeled with an F-actin binding peptide known as Lifeact (Movies S1 and S2; Figure 6A) (Riedl et al., 2008). Upon infection, both WT bacteria and *S. flexneri* Δ*ipaH9.8* became coated with GBP1, a status that, once established, was sustained throughout division by bacteria of either strain. However, while *S. flexneri* Δ*ipaH9.8* maintained their GBP1 coat over the time course of the movie, i.e., for at least 3 hr, WT bacteria lost their coat between 1 and 1.5 hr p.i., followed by the acquisition of actin-dependent motility and invasion of neighboring cells, where sometimes *de novo* GBP coating occurred. In contrast, *S. flexneri* Δ*ipaH9.8*, due to lack of actin-driven motility, formed tightly clustered micro-colonies and failed to invade neighboring cells. Quantification confirmed the absence of GBP1-positive bacteria displaying actin tails in WT and Δ*ipaH9.8* strains, as well as the lower and higher percentage of actin tails and GBP1 coats, respectively, in *S. flexneri* Δ*ipaH9.8* compared to WT bacteria (Figure 6B). Complementation with *ipaH9*.*8* reversed the actin phenotype (Figure 6C) and treatment with the proteasome inhibitor Carfilzomib antagonized the IpaH9.8 effect on actin tail formation (Figure S5). To test whether GBPs mediate the IFNγ-induced suppression of actin tail formation, we depleted cells of GBPs using small interfering RNAs (siRNAs) (Figure 6D) and discovered that GBP1 was essential for IFNγ to antagonize actin tail formation on *S. flexneri*. Finally, to investigate the functional consequences of IpaH9.8-mediated degradation of IFNγ-induced GBPs and its effect on actin tail formation, we measured proliferation of *S. flexneri* and bacterial load in host cells. Bacterial proliferation in either resting or IFNγ-treated cells was unaffected by deletion of IpaH9.8 (Figure 6E). However, bacteria lacking IpaH9.8 did not spread efficiently between cells, resulting in a much smaller number of cells becoming infected (Figures 6F, 6G, and S7) and those cells carrying a much higher bacterial load (Figures 6F, 6H, and S7). Taken together, we conclude that GBPs restrict actin-driven motility of *S. flexneri*, thus impairing the spread of bacteria into neighboring, uninfected cells and containing the bacterial burden in a smaller number of infected cells. IpaH9.8 antagonizes GBP-mediated cell-autonomous immunity by targeting GBPs for proteasomal degradation, resulting in unrestricted spread of bacteria.

## Discussion

GBPs provide important but poorly understood immunity against invasive bacteria in interferon-stimulated cells. Here we provide evidence for the GBP1-dependent hierarchical recruitment of multiple GBPs to cytosol-exposed *S. flexneri*, where they form a dense GBP coat surrounding the bacterium that inhibits actin-dependent bacterial motility and, consequently, cell-to-cell spread. *S. flexneri* antagonizes GBP-mediated cellular defenses by secreting the E3 ubiquitin ligase IpaH9.8, which ubiquitylates and degrades GBPs in a proteasome-dependent manner, causing existing GBP coats to dissolve and actin-dependent motility to become re-established. The existence of an E3 ubiquitin ligase in the genome of *S. flexneri* that efficiently degrades GBPs provides compelling evolutionary evidence for the importance of GBPs in anti-bacterial defense.

GBPs clearly play an important role in defending the cell interior against parasites and bacteria, although the target of GBP attack and their mode of action remain poorly characterized (Kim et al., 2012, Man et al., 2017). While both host membranes and bacterial surfaces have been found to be targeted by GBPs, our SIM super-resolution data provide unequivocal evidence for the accumulation of GBPs on the bacterial surface with no observable enrichment on galectin-8-positive remnants of *Shigella*-containing vacuoles. Importantly, the accumulation of GBPs on *S. flexneri* required catalytically active GBP1, suggesting a clear hierarchy in GBP recruitment and the possibility that GBP1 either serves as a receptor for *S. flexneri* and possibly other pathogens or that other cellular proteins are required to load GBPs in a hierarchical manner onto the bacterial surface. Similar to the ubiquitin coat formed by cellular E3 ubiquitin ligases when they encounter bacteria in the host cytosol (Franco et al., 2017, Huett et al., 2012, Manzanillo et al., 2013, Noad et al., 2017, Perrin et al., 2004), the GBP coat also represents a highly polyvalent display of host proteins on the bacterial surface. Therefore, just like the ubiquitin coat, the GBP coat may also transform the bacterial surface into a signaling platform (Noad et al., 2017, van Wijk et al., 2017). Considering the time required to upregulate IFNγ-dependent GBP expression and the model character of the cells used in this study, follow-up investigations will be required to test the importance of the GBP coat for the defense against *S. flexneri in vivo*.

While several effector molecules of the ubiquitin coat have been identified, including pro-inflammatory signaling molecules such as the Nemo-recruited IKK complex (Noad et al., 2017) and autophagy cargo receptors such as NDP52, Optineurin, and p62 (Thurston et al., 2009, Wild et al., 2011, Zheng et al., 2009), GBP effectors remain to be identified, although IRGB10, recruited to *Francisella novicida* via GBPs and contributing to the liberation of bacterial ligands for the inflammasome, may represent an important effector molecule of the GBP coat (Man et al., 2016).

Since the bacterial ubiquitin coat serves anti-bacterial purposes, *S. flexneri*, as a professional cytosol-dwelling pathogen, has evolved sophisticated countermeasures against ubiquitin coating, such as, for example, degrading anti-bacterial E3 ligases (de Jong et al., 2016, Noad et al., 2017). However, in cells stimulated with IFNγ, *S. flexneri* does become ubiquitin coated, although not due to the action of cellular E3 ubiquitin ligases, but as a result of IpaH9.8 ubiquitylating the GBP coat. Therefore, rather than promoting anti-bacterial defense mechanisms by providing “eat-me” signals for cargo receptors of xenophagy (Boyle and Randow, 2013), the coating of *S. flexneri* with polyubiquitin by IpaH9.8 results from the bacterial attempt to counteract a cellular defense pathway. Care must therefore be taken when attempting to assign anti-bacterial function to the ubiquitin coat on cytosolic bacteria. Our results on the ubiquitin coating of *S. flexneri* by a bacterially encoded E3 ubiquitin ligase are congruent with a growing body of literature suggesting that *S. flexneri* possesses multiple mechanisms to evade anti-bacterial autophagy. Previous studies have shown that Toca1 recruitment to intracellular *S. flexneri* antagonizes xenophagy and that IcsB and VirA are bacterially encoded factors that help in evading anti-bacterial autophagy (Baxt and Goldberg, 2014, Campbell-Valois et al., 2015, Ogawa et al., 2005).

IpaH9.8 is an important pathogenicity factor for *S. flexneri* since, in a murine pneumonia model, *S. flexneri* Δ*ipaH9.8* proliferates less and induces higher levels of pro-inflammatory cytokines (Okuda et al., 2005). Degradation of both U2AF35, a splicing factor, and Nemo, an essential subunit of the NF-κB-inducing IKK complex, has been suggested to cause the observed phenotype (Ashida et al., 2010, Okuda et al., 2005). Our work revealed that IpaH9.8, among a panel of twelve IpaH proteins, specifically targets GBP1, GBP2, and GBP4, but not GBP3. As demonstrated for GBP1 and GBP3, binding of GBPs to IpaH9.8 parallels the ability of IpaH9.8 to ubiquitylate GBPs *in vitro*, consistent with the autoinhibited state of IpaH E3 ligases in the absence of bound substrate (Chou et al., 2012), and the ability of IpaH9.8 to induce proteasome-dependent degradation in cells. GBP degradation does not require GBP oligomerization on the bacterial surface, since expression of IpaH9.8 in uninfected cells was sufficient to deplete GBP from cells, suggesting sufficiently high affinity of IpaH9.8 for soluble GBPs consistent with binding of IpaH9.8 to GBP1, GBP2, and GBP4 in LUMIER interaction assays. The ability of IpaH9.8 and potentially other IpaH proteins to selectively degrade key host defense molecules suggests that IpaH proteins hold significant potential for the experimental manipulation of host-pathogen interactions, although it will be important to first gain a better understanding of the structural basis of IpaH specificity.

## STAR★Methods

### Key Resources Table

**Table:** 

REAGENT or RESOURCE	SOURCE	IDENTIFIER
**Antibodies**		

GFP (JL8) mouse monoclonal	Clontech	Cat# 632381; RRID: AB_2313808
Ubiquitin (FK2) mouse monoclonal	Enzo Life Science	Cat# BML-PW8810; RRID: AB_10541840
Ubiquitin M1 (1E3) rabbit monoclonal	Merck Millipore	Cat# MABS199; RRID: AB_2576212
Ubiquitin K11 (2A3/2E6) rabbit monoclonal	Merck Millipore	Cat# MABS107-I; RRID: AB_2713901
Ubiquitin K48 (Apu2) rabbit monoclonal	Merck Millipore	Cat# 05-1307; RRID: AB_1587578
PCNA (PC10) mouse monoclonal	Santa Cruz Biotechnology	Cat# sc-56; RRID: AB_628110
Galectin-8 goat polyclonal	R&D Systems	Cat# AF1305; RRID: AB_2137229
GBP1 rabbit polyclonal	Proteintech	Cat# 15303-1-AP; RRID: AB_2247448
GBP1 (1B1) rat monoclonal	Santa Cruz Biotechnology	Cat# sc-53857; RRID: AB_2109333
GBP1-5 (G-12) mouse monoclonal	Santa Cruz Biotechnology	Cat# sc-166960; RRID: AB_10611378
GBP2 (G-9) mouse monoclonal	Santa Cruz Biotechnology	Cat# sc-271568; RRID: AB_10655677
Ubiquitin K27 rabbit monoclonal	Abcam	Cat# ab181537; RRID: AB_2713902
Flag-tag (M2) mouse monoclonal	Sigma	Cat# F1804; RRID: AB_262044
β-actin rabbit polyclonal	Abcam	Cat# ab8227; RRID: AB_2305186
Alexa-conjugated anti-mouse	Thermo Fisher Scientific	N/A
Alexa-conjugated anti-rabbit	Thermo Fisher Scientific	N/A
HRP-conjugated reagents	Dabco	N/A

**Bacterial and Virus Strains**		

*Shigella flexneri* strain M90T	Gift from Chris Tang	N/A
*Shigella flexneri* strain M90T Δ*icsA*	Gift from Chris Tang	N/A
*Shigella flexneri* strain M90T Δ*mxiE*	Sidik et al., 2014	N/A
*Shigella flexneri* strain M90T Δ*ipaH1.4*	Sidik et al., 2014	N/A
*Shigella flexneri* strain M90T Δ*ipaH7.8*	Sidik et al., 2014	N/A
*Shigella flexneri* strain M90T Δ*ipaH9.8*	Sidik et al., 2014	N/A
*Salmonella* Typhimurium strain 12023	Gift from David Holden	N/A

**Chemicals, Peptides, and Recombinant Proteins**		

Phalloidin Alexa488	Thermo Fisher Scientific	Cat# A12379
Phalloidin Alexa568	Thermo Fisher Scientific	Cat# A12380
Human IFNγ	R&D Systems	Cat# 285-IF
Human IFNβ	PBL	Cat# 11415-1
Human TNFα	R&D Systems	Cat# 210-TA
Human IL1-β	R&D Systems	Cat# 201-LB
Human IL-22	R&D Systems	Cat# 782-IL
Lipofectamine RNAiMAX	Thermo Fisher Scientific	Cat# 13778150
VECTASHIELD HardSet Antifade Mounting Medium with DAPI	Vector Laboratories	Cat# H-1500
ProLong Gold Antifade Mountant	Thermo Fisher Scientific	Cat# P36930
Carfilzomib (PR-171)	Selleck Chemicals	Cat# S2853
Polyethylenimine (PEI)	Polysciences	Cat# 23966-2
Saponine	Thermo Fisher Scientific	Cat# AC419231000
E1 enzyme (UB reagent)	Gift from Paul Elliott, MRC LMB Cambridge	N/A
UBE2D1 (UB reagent)	Gift from Paul Elliott, MRC LMB Cambridge	N/A
Ubiquitin (UB reagent)	Gift from Paul Elliott, MRC LMB Cambridge	N/A
cOmplete Protease Inhibitor Cocktail	Roche	Cat#000000011697498001

**Critical Commercial Assays**		

*Renilla* Luciferase Assay System	Promega	Cat# E2820
Fix & Perm	Thermo Fisher Scientific	Cat# GAS004
RNeasy Plus Mini Kit	QIAGEN	Cat# 74134
Amersham ECL	GE Healthcare Life Sciences	Cat# RPN2106

**Deposited Data**		

DNA microarray	This study	GEO: GSE103363

**Experimental Models: Cell Lines**		

HeLa	Lab strain	N/A
293ET	Lab strain	N/A
THP1	Lab strain	N/A

**Oligonucleotides**		

See Table S2 for siRNAs	This study	N/A

**Recombinant DNA**		

pFPV25.1 *ipaH9.8*	This study	CP75
pFPV25.1 *ipaH9.8* C337A	This study	CP76
M6P GFP::*ipaH1.4* C368A	This study	CP37
M6P GFP::*ipaH2.5* C368A	This study	CP38
M6P GFP::*ipaH4.5* C379A	This study	CP39
M6P GFP::*ipaH7.8* C357A	This study	CP40
M6P GFP::*ipaH9.8* C337A	This study	CP41
M6P GFP::*ipaH1* C379A	This study	CP48
M6P GFP::*ipaH2* C400A	This study	CP42
M6P GFP::*ipaH3* C340A	This study	CP43
M6P GFP::*ipaH4* C375A	This study	CP44
M6P GFP::*ipaH5* C339A	This study	CP45
M6P GFP::*ipaH6* C339A	This study	CP46
M6P GFP::*ipaH7* C400A	This study	CP47
M6P Flag::*ipaH1.4*	This study	CP02
M6P Flag::*ipaH2.5*	This study	CP03
M6P Flag::*ipaH4.5*	This study	CP04
M6P Flag::*ipaH7.8*	This study	CP05
M6P Flag::*ipaH9.8*	This study	CP06
M6P Flag::*ipaH2*	This study	CP07
M6P Flag::*ipaH3*	This study	CP08
M6P Flag::*ipaH4*	This study	CP09
M6P Flag::*ipaH5*	This study	CP10
M6P Flag::*ipaH6*	This study	CP11
M6P Flag::*ipaH7*	This study	CP12
M6P Flag::*ipaH1*	This study	CP13
M6P Flag::*ipaH9.8* C337A	This study	CP67
pETM30 HIS::GST::*ipaH9.8*	This study	CP77
M6P GFP::*GBP1*	This study	MW478
M6P GFP::*GBP2*	This study	MW479
M6P GFP::*GBP3*	This study	MW480
M6P GFP::*GBP4*	This study	MW482
M6P GFP::*GBP5*	This study	MW483
M6P GFP::*GBP6*	This study	MW484
M6P GFP::*GBP7*	This study	MW485
M6P GFP::*GBP1* R48A	This study	MW562
M6P GFP::*GBP1* K51A	This study	MW564
M6P GFP::*GBP1* S52N	This study	MW566
M6P GFP::*GBP1* D184N	This study	MW567
pETM11 His_6_::*GBP1*	This study	MW594
pETM11 His_6_::*GBP3*	This study	MW596
M5P luciferase::*GBP1*	This study	MW544
M5P luciferase::*GBP2*	This study	MW545
M5P luciferase::*GBP3*	This study	MW546
M5P luciferase::*GBP4*	This study	MW548
M6P YFP::*Galectin-8*	This study	MW319
M6P *N-WASP*::GFP	This study	MW925
M6P GFP::*WIP*	This study	MW919
M6P *ARP2*::GFP	This study	MW926
M6P GFP::*ARP3*	This study	MW923
M6P *STAT1*::GFP	This study	MW380
M6P *Lifeact*::CFP	This study	MW918
pFPV25.1 FLAG::*icsA*	This study	MW933
M6P GFP::*RAP80UIM* (K63 probe)	This study	AL96

**Software and Algorithms**		

GraphPad Prism	https://www.graphpad.com/scientific-software/prism/	N/A
FlowJo	https://www.flowjo.com	N/A
Zeiss ZEN	https://www.zeiss.com/microscopy/int/products/microscope-software/zen-lite.html	N/A
aCOLyte3	http://www.synbiosis.com/acolyte-software/	N/A

### Contact for Reagent and Resource Sharing

Further information and requests for resources and reagents should be directed to and will be fulfilled by the Lead Contact, Felix Randow (randow@mrc-lmb.cam.ac.uk).

### Experimental Model and Subject Details

#### Cell Culture

HeLa, 293ET and THP1 cells, as well as all stable cell lines, were grown in IMDM supplemented with 10% FCS at 37c°C in 5% CO2. HeLa and 293ET are of female, THP1 of male origin. Cell lines have not been authenticated. All cell lines were tested to be Mycoplasma free.

#### Bacteria

*S. flexneri* (strain M90T) was grown in tryptic soy broth (TSB) or on tryptic soy agar containing 0.003% Congo red.

*S*. Typhimurium (strain 12023) was grown in Luria broth (LB) or on LB agar plates.

*E. coli* strains MC1061, BL21 and Rosetta2 were grown on tryptic yeast extract (TYE) agar plates or in LB.

### Method Details

#### Cytokine Treatment

Cytokines were added for 10-20 hours before experiments at the following concentrations: IFNγ 1 ng/ml, IFNβ 100 U/ml, TNFα 10 ng/ml, IL1-β 10 ng/ml and IL-22 10 ng/ml.

#### Plasmid Generation

M5P or closely related plasmids were used to produce recombinant MLV for the expression of proteins in mammalian cells (Randow and Sale, 2006). Open reading frames encoding IpaH proteins, N-WASP, WIP, ARP2, ARP3 and GBP1-7 were amplified by PCR. Mutations were generated by PCR and verified by sequencing. For complementation IpaH9.8 and FLAG::IcsA (Mauricio et al., 2017) were amplified by PCR and cloned into pFPV25.1.

#### Bacterial Infections and Enumeration of Intracellular Bacteria

*S. flexneri* was grown overnight in tryptic soy broth (TSB) and sub-cultured (1:100) in fresh TSB for 2.5 h before infection. Such cultures were consecutively washed in PBS and re-suspended in antibiotic-free IMDM plus 10% FCS immediately before 100 μL was used to infect HeLa cells in 24-well plates. Samples were centrifuged for 10 min at 670 g followed by incubation at 37°C for 20 or 30 min. Following two washes with warm PBS, cells were cultured in 100 μg/ml gentamycin for 2 h and 20 μg/ml gentamycin thereafter. For FACS-based infection assays, bacteria were diluted 1:3 in warm antibiotic-free IMDM before infection and cells were washed 10 min after infection.

*S*. Typhimurium was grown overnight in Luria broth (LB) and sub-cultured (1:33) in fresh LB for 3.5 h before infection. Such cultures were further diluted (1:5) in antibiotic-free IMDM plus 10% FCS immediately before 20 μL was used to infect HeLa cells in 24-well plates for 15 min at 37°C. Following two washes with warm PBS, cells were cultured in 100 μg/ml gentamycin.

To enumerate intracellular bacteria, cells from triplicate wells were lysed in 1 ml cold PBS containing 0.1% Triton X-100. Serial dilutions were plated in duplicate on TYE agar.

#### Microarray

Total RNA from HeLa cells was extracted using the RNeasy Plus Mini Kit. RNA samples were prepared using the Ambion WT Expression Kit and Affymetrix GeneChip WT Terminal Labeling and Hybridization Kit. The generated cocktails were hybridized to Human Affymetrix Gene ST 1.0 cartridge arrays. The arrays were then scanned on the Affymetrix GCS3000 by the Addenbrooke’s Hospital Genomics Core Laboratory.

#### RNA Interference

5 × 10^4^ cells per well were seeded in 24-well plates. The following day, cells were transfected with 40 pmol of siRNA using Lipofectamine RNAiMAX. Experiments were performed after 3 days.

The non-targeting negative control was used as control.

#### Microscopy

HeLa cells were grown on glass coverslips before infection. After infection, cells were washed twice with warm PBS and fixed in 4% paraformaldehyde for 20 min. Cells were washed twice in PBS and then simultaneously permeabilized and blocked in PBSB (PBS, 0.01% saponin, 2% BSA). Coverslips were incubated with primary followed by secondary antibodies for 1 h in PBSB. Samples were mounted either in mounting medium with DAPI or Prolong Antifade mounting medium for confocal imaging and super resolution microscopy, respectively. Marker positive bacteria were scored by eye among at least 200 bacteria per coverslip. Confocal images were taken with a × 63, 1.4 numerical aperture objective on either a Zeiss 710 or a Zeiss 780 microscope. Live imaging was performed on a Nikon Eclipse Ti equipped with an Andor Revolution XD system and a Yokogawa CSU-X1 spinning disk unit. Super resolution images were acquired using an Elyra S1 structured illumination microscope (Carl Zeiss Microscopy Ltd, Cambridge, UK). The system has four laser excitation sources (405nm, 488nm, 561nm and 640nm) with fluorescence emission filter sets matched to these wavelengths. SIM Images were obtained using a 63X 1.4 NA oil immersion lens with grating projections at 3 rotations and 5 phases in accordance with the manufacturers instructions. The number of Z planes varied with sample thickness. Super resolution images were calculated from the raw data using Zeiss ZEN software.

#### Immunoprecipitation and Western Blot

293ET cells were grown on 6-well plates and transfected with 2 μg plasmid DNA (1.5 μg GFP:GBP1 and 0.5 μg IpaH9.8) using PEI. 24h after transfection the proteasome was inhibited for 18h using 100 nM Carfilzomib.

Post-nuclear supernatants from 2 × 10^6^ 293ET cells expressing tagged proteins were obtained following lysis (150 mM NaCl, 0.1% Triton X-100, 20 mM Tris-HCl pH 7.4, 5 mM EDTA and proteinase inhibitors). Proteins were precipitated for 2 h with anti-GFP or Flag agarose before washing. Samples were eluted with Flag peptide or Laemmli buffer and separated on 4%–12% denaturing Bis-Tris gels (Thermo Fisher Scientific). Visualization following immunoblotting was performed using ECL detection reagents.

#### Protein Expression for LUMIER Assays

Proteins were expressed from pETM30 in *E.coli* BL21. Bacteria were grown to an OD_600_ of 0.7 at 37°C before overnight induction at 16°C in the presence of 100 μM IPTG. Cells were mechanically lysed in lysis buffer (20mM Tris (pH 8.0), 150mM NaCl, 1mM DTT, protease inhibitors) and cleared by centrifugation. Lysates were snap frozen and stored at −80°C.

#### LUMIER Assays

LUMIER binding assays with pairs of putative interactors, one fused to luciferase and the other fused to GST, were performed in LUMIER lysis buffer (150 mM NaCl, 0.1% Triton X-100, 20 mM Tris-HCl (pH 7.4), 5% glycerol, 5 mM EDTA and proteinase inhibitors) as previously described (Ryzhakov and Randow, 2007). GST-fusion proteins were immobilized on beads before incubation with the luciferase tagged binding partner for 2 h. Luciferase-tagged proteins were expressed in 293ET cells. After washing in lysis buffer, proteins were eluted with glutathione in *Renilla* lysis buffer (Promega). Relative luciferase activity represents the ratio of activity eluted from beads and present in lysates.

#### Protein Expression and Purification for *In Vitro* Ubiquitination Assay

His_6_-GBP1 and His_6_-GBP3 (both in the pETM-11 vector) and His_6_-GST-IpaH9.8 (in pETM-30 vector) were expressed in Rosetta2 (DE3) cells. Cells were grown at 30°C in 2xTY medium supplemented with 30 μg/ml kanamycin and 34 μg/ml chloramphenicol. Cultures were induced with IPTG (400 μM) at 18°C and harvested after 16 hours. Proteins were purified by immobilized metal-affinity chromatography and the His_6_-GST tag was removed from IpaH9.8 by incubation with TEV protease. His_6_-GBP1, His_6_-GBP3 and untagged IpaH9.8 were further purified by anion exchange chromatography using a Resource Q column (GE Healthcare) and size exclusion chromatography using a Superdex 200 16/60 column (GE Healthcare), the latter in buffer 20 mM Tris pH 8.5, 200 mM NaCl and 4 mM DTT.

#### *In Vitro* Ubiquitination Assay

Reactions were set up in 40 mM Tris pH 8.5, 10 mM MgCl_2_, 0.6 mM DTT with combinations of the following reagents: E1 (0.5 μM), UBE2D1 (3 μM), IpaH9.8 (5 μM), His_6_-GBP1 (30 μM), His_6_-GBP3 (30 μM), ubiquitin (300 μM) and ATP (10 mM). The reaction was incubated at 37°C for 30 min after which, Ni^II^ resin was added for 15 minutes at r.t.. Following binding, resin was washed three times in 20 mM Tris pH 7.4, 300 mM NaCl, 50 mM Imidazole, 2 mM β-mercaptoethanol and 0.01% tween.

#### FACS

After infection, cells were washed twice with warm PBS, detached with trypsin and fixed in 4% paraformaldehyde for 20 min. Cells were washed twice in PBS and quenched with PBSG (PBS, 1M Glycine). For intracellular staining samples were fixed and stained using Fix & Perm (Life Technologies). All samples were analyzed on a BD LSR ii flow cytometer, using the high throughput system (HTS) to score spread of bacteria. Data were analyzed in FlowJo.

#### Transformation of *S. flexneri*

A Tryptic Soy Broth (TSB) overnight culture was diluted 1:100 in 10 mL of TSB and grown at 37°C to an OD600 of 0.6 to 0.8. Bacteria were cooled on ice for 10 min, centrifuged (4,300 g x 4 min, 4 C) and washed once in 10 mL then twice in 1 mL of ice cold electroporation buffer (1 mM MOPS, 20% glycerol, pH 7.2). Bacteria were pelleted a final time, resuspended in 100 μL of buffer and mixed with 150 ng of plasmid DNA. The mixture was electroporated in a chilled 2mm cuvette (Flowgen Bioscience) using 2,500V, 600 Ω and 10 μF. Electroporated bacteria were regenerated for 1 h at 37 C in 1 mL of Super Optimal Broth (SOB) medium and plated on TSB agar supplemented with Ampicillin (100 μg/mL) and Congo red (0.003%).

### Quantification and Statistical Analysis

All data were tested for statistical significance with Prism software (GraphPad Prism 7). The unpaired Student’s t test was used to test whether two samples originate from the same population. For more than two samples with only one variable an analysis of variance (one-way ANOVA) was performed. Either Dunnett’s multiple comparison test (to compare all samples against a control) or Tukey’s multiple comparison test (to compare all samples against each other) was applied. Performed tests are indicated in Figure Legends. No specific method was used to determine whether the data met assumptions of the statistical approach. Unless otherwise stated, all experiments were performed at least three times and the data were combined for presentation as mean ± SEM. All differences not specifically indicated as significant were not significant (ns, p > 0.05). Significant value are indicated as ^∗^, p < 0.05; ^∗∗^, p < 0.01. Statistical details, including sample size (n), are reported in the Figures and Figure Legends.

#### Microscopy

For scoring marker positive bacteria three independent experiments with three replicates each were performed. Bacteria were scored by visual enumeration as n > 100 (for 1h p.i.), n > 200 (for 2h p.i.) or n > 300 (for 3h p.i.) bacteria per replicate. Graphs show mean ± SEM.

#### Scoring Intracellular Bacteria

To score bacterial burdens, cells from triplicate wells were lysed and bacteria were plated in duplicate on TYE agar. Each experiment was performed three times. Bacterial colonies were counted using the aCOLyte3 system (Synbiosis).

Graphs show mean ± SD of a representative experiment or mean ± SEM for combined datasets.

### Data and Software Availability

The accession number for the DNA microarray data reported in this paper is NCBI Gene Expression Omnibus, GEO: GSE103363.

## Author Contributions

M.P.W., C.P., E.I.W., C.J.E., K.B.B., and A.v.d.M. performed experiments and analyzed data. J.R. provided knockout strains and reviewed the manuscript. M.P.W., C.P., and F.R. wrote the manuscript.

## Supplemental Information

Document S1. Figures S1–S6 and Table S2

Table S1. Microarray Analysis of Interferon-Regulated Genes, Related to Figure 2 Expression values (log2) for genes in control (untreated), IFNγ or IFNβ treated HeLa cells and fold change in gene expression for interferon-treated samples versus control. Genes upregulated over 2 fold in IFNγ versus IFNβ treated sample are shown.

Movie S1. Wild-Type *S. flexneri* Develop Actin Tails and Become Motile Once Their GBP1 Coat Has Been Degraded, Related to Figure 6 Live imaging on a confocal spinning disk microscope of cells expressing GFP::GBP1 and Lifeact::CFP, infected with Ruby-expressing *S. flexneri* and imaged every 2 min. Scale bar 10 μm.

Movie S2. *S. flexneri* Δ*ipaH9.8* Do Not Degrade Their GBP1 Coat and Remain Immotile, Related to Figure 6 Live imaging on a confocal spinning disk microscope of cells expressing GFP::GBP1 and Lifeact::CFP, infected with Ruby-expressing *S. flexneri* Δ*ipaH9.8* and imaged every 2 min. Scale bar 10 μm.

Document S2. Article plus Supplemental Information
