# Supplementary material for: GBPs Inhibit Motility of Shigella flexneri but Are Targeted for Degradation by the Bacterial Ubiquitin Ligase IpaH9.8
Source: Cell Host Microbe. 2017 Oct 11;22(4):507–518.e5. doi: 10.1016/j.chom.2017.09.007 (PMC5644667; doi:10.1016/j.chom.2017.09.007)
Supplement: Document S1. Figures S1–S6 and Table S2 [file mmc1.pdf]

Cell Host & Microbe, Volume *22*

Supplemental Information

GBPs Inhibit Motility of *Shigella flexneri* but Are Targeted for Degradation by the Bacterial Ubiquitin Ligase IpaH9.8

Michal P. Wandel, Claudio Pathe, Emma I. Werner, Cara J. Ellison, Keith B. Boyle, Alexander von der Malsburg, John Rohde, and Felix Randow

Supplementary figure 1: Validation of siRNAs, Related to Figure 2

(A-B) Lysates from HeLa cells expressing GFP-tagged **(A)** STAT1 or **(B)** GFP-tagged GBP1-4 treated with the indicated siRNAs against **(A)** STAT1 or **(B)** GBP1-4. Blots were probed with GFP and PCNA antibodies.

Supplementary Figure 1

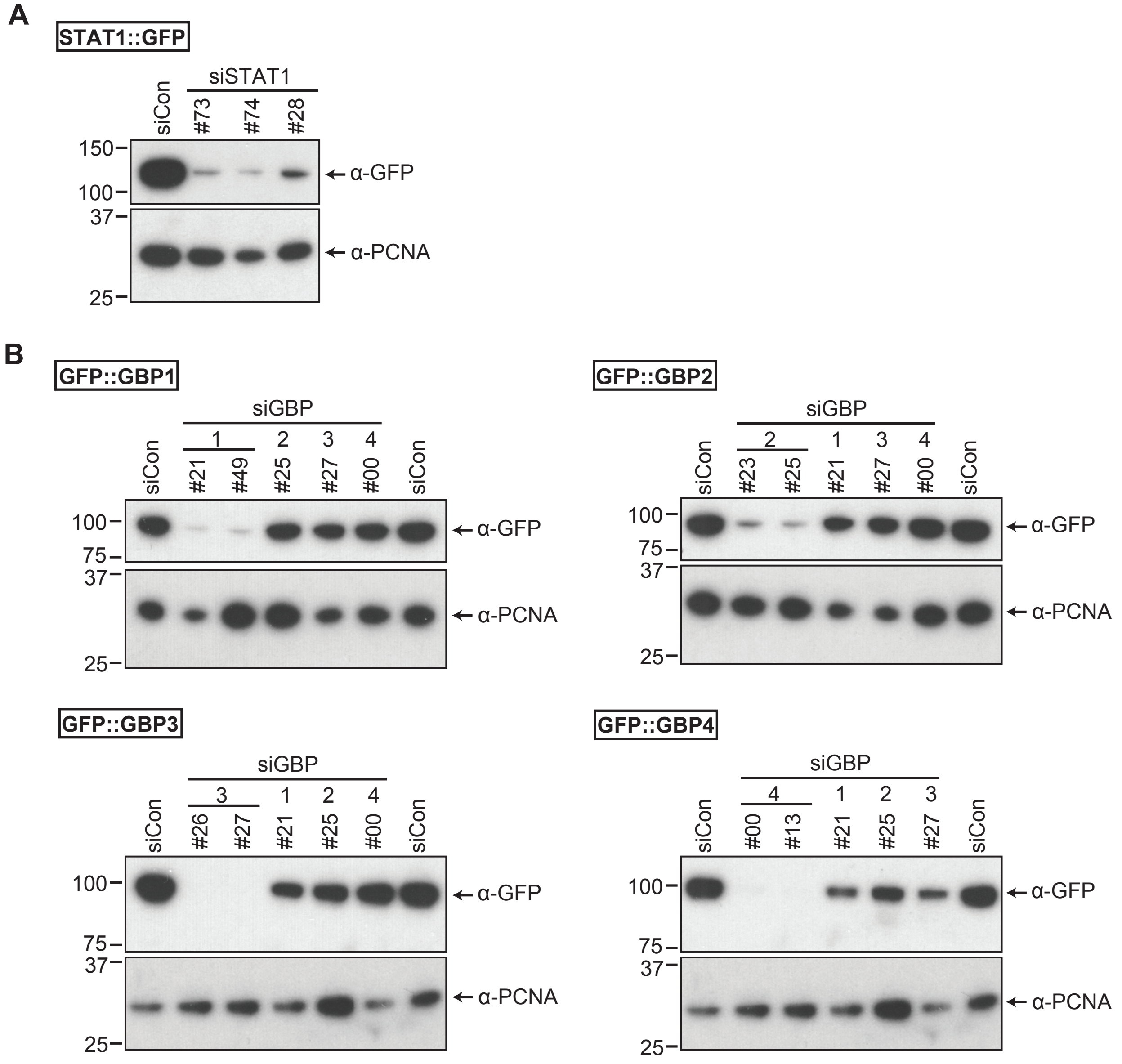

Supplementary figure 2: Recruitment of GBPs to *S. flexneri,* Related to Figure 2

Confocal micrographs of HeLa cells expressing GFP-tagged GBPs, treated with IFNγ and infected with *S. flexneri*. Images taken at 1h p.i., after staining for Galectin-8. Scale bar 10μm.

Supplementary Figure 2

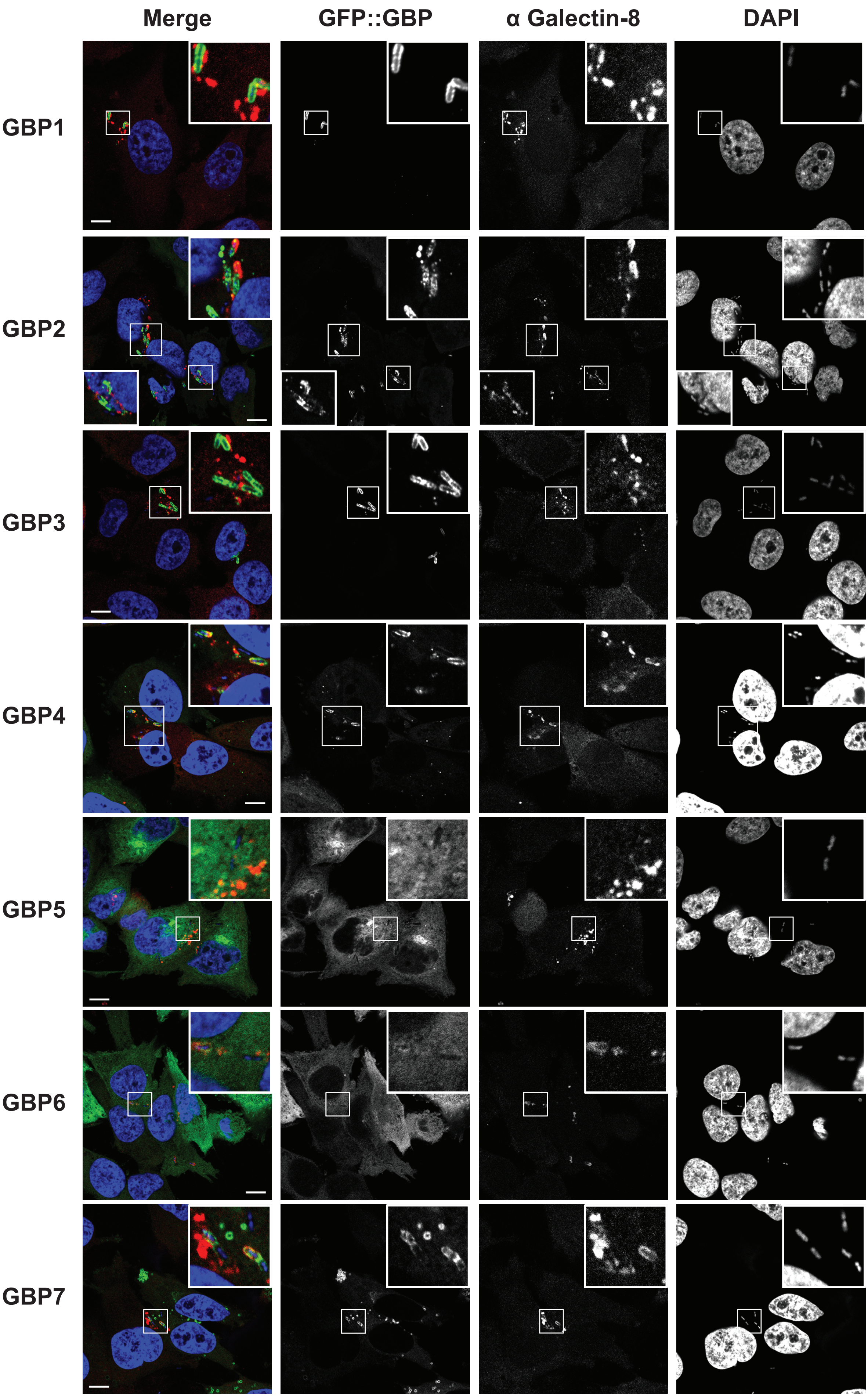

Supplementary figure 3: Co-localisation of GBP1-4 and K48-linked ubiquitin chains on *S. flexneri,* Related to Figure 2

Confocal micrographs of HeLa cells expressing GFP-tagged GBP1-4 treated with IFNγ and infected with *S. flexneri*. Images taken at 2h p.i. after staining for K48-linked ubiquitin. Scale bar 10µm.

Supplementary Figure 3

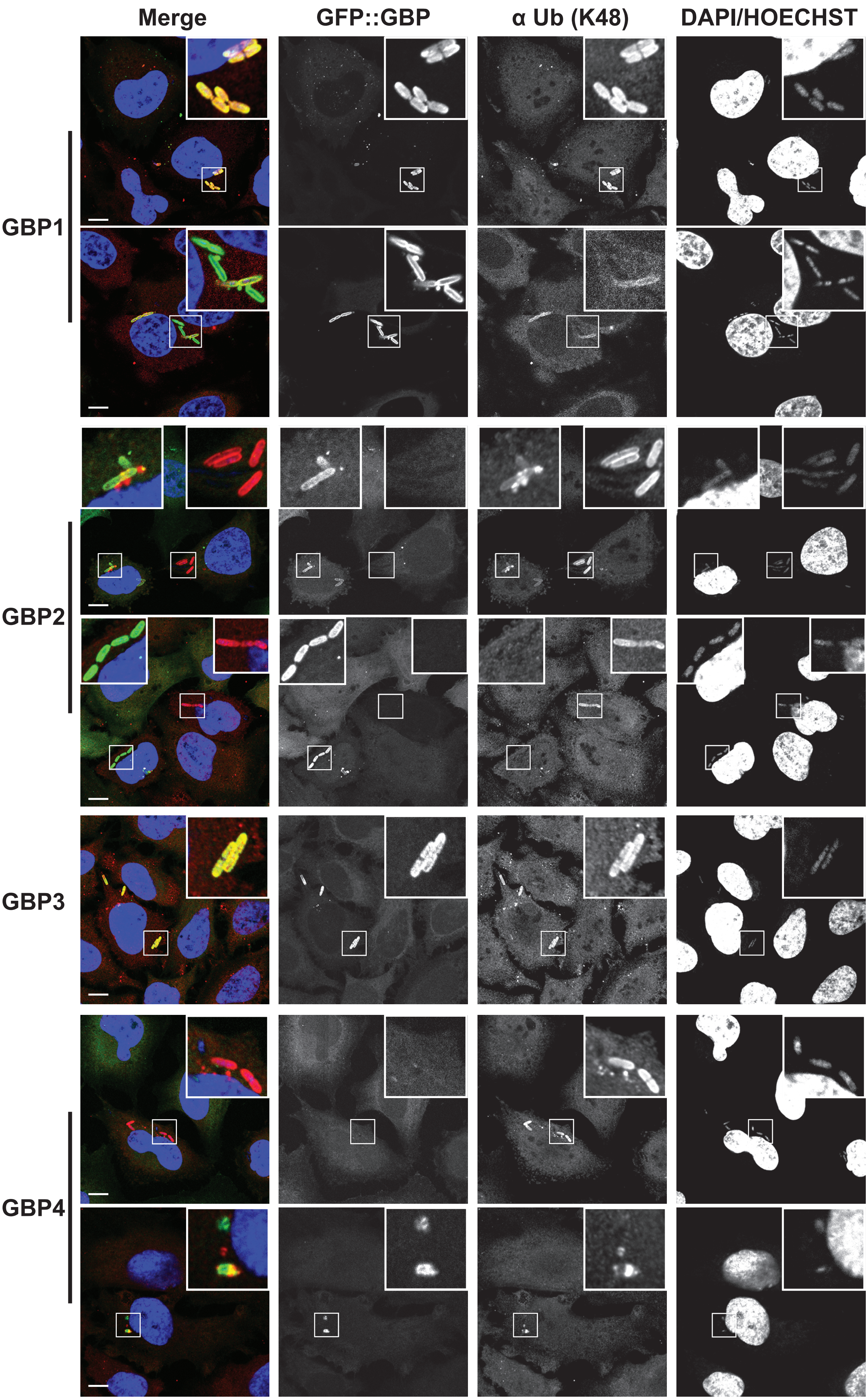

Supplementary figure 4: IpaH9.8 degrades cellular GBPs, Related to Figure 4

Gating strategy for Fig.4B. Representative dotplots of control or HeLa cells expressing GFP:GBP1-4, infected with Ruby-expressing *S. flexneri* for 10 or 180 min and gated for intracellular bacteria (large gate). Infected cells were sub-gated for high or low expression levels of GBPs. Percentages are indicated for infected cells (top) and percentage of infected cells with low levels of GBPs (bottom).

Supplementary Figure 4

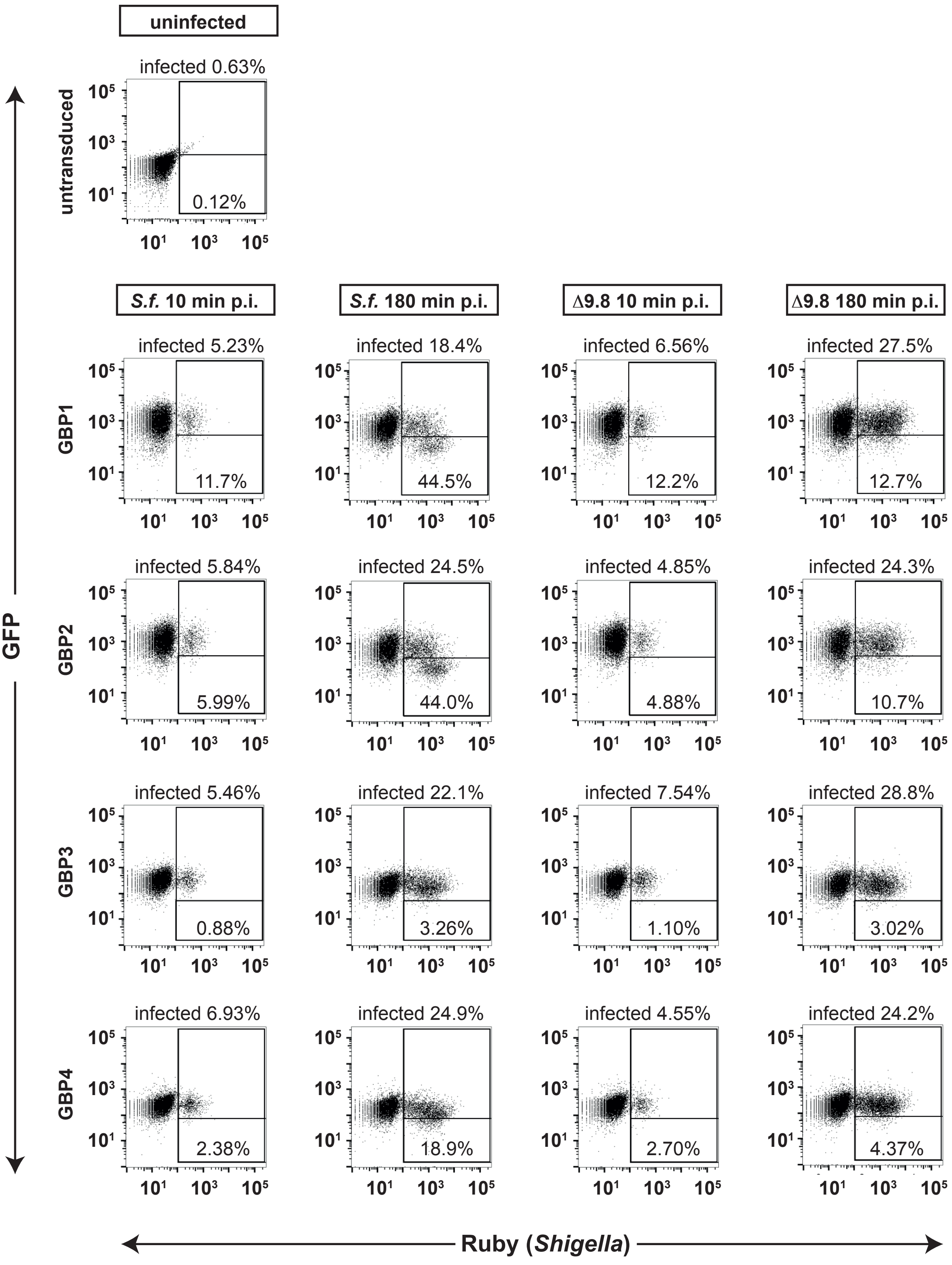

Supplementary figure 5: Carfilzomib antagonizes the IpaH9.8 effect on actin tail formation, Related to Figure 6

S. flexneri positive for actin tails at 2h p.i. in HeLa cells stimulated with IFNγ as indicated. 100nM Carfilzomib or DMSO was added 30 min p.i. and kept throughout the infection. Mean±SEM of triplicate coverslips from three independent repeats, n>200 bacteria per coverslip. ns=not significant, **P<0.01 one-way ANOVA with Tukey's multiple comparisons test.

Supplementary Figure 5

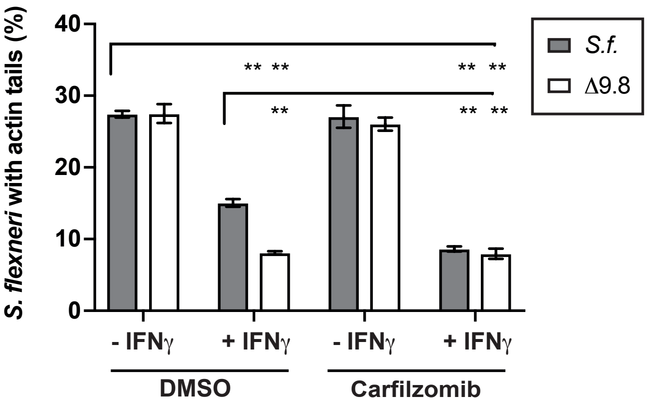

Supplementary figure 6: FACS analysis of bacterial load and cell-to-cell spread of *S. flexneri* Δ*ipaH9.8,* Related to Figure 6

HeLa cells were infected with the indicated *S. flexneri* strains expressing Ruby in the presence or absence of IFNγ for the indicated times. Cells were analysed by flow cytometry and gated for infected cells (red). Infected cells were gated for high bacterial load (black gate). Histograms show infected cells at 10 min (black), 120 min (blue) and 300 min (red) with gate for high levels of Ruby (black line). Plots and histograms are a representative dataset of Figure 6G,H.

Supplementary Figure 6

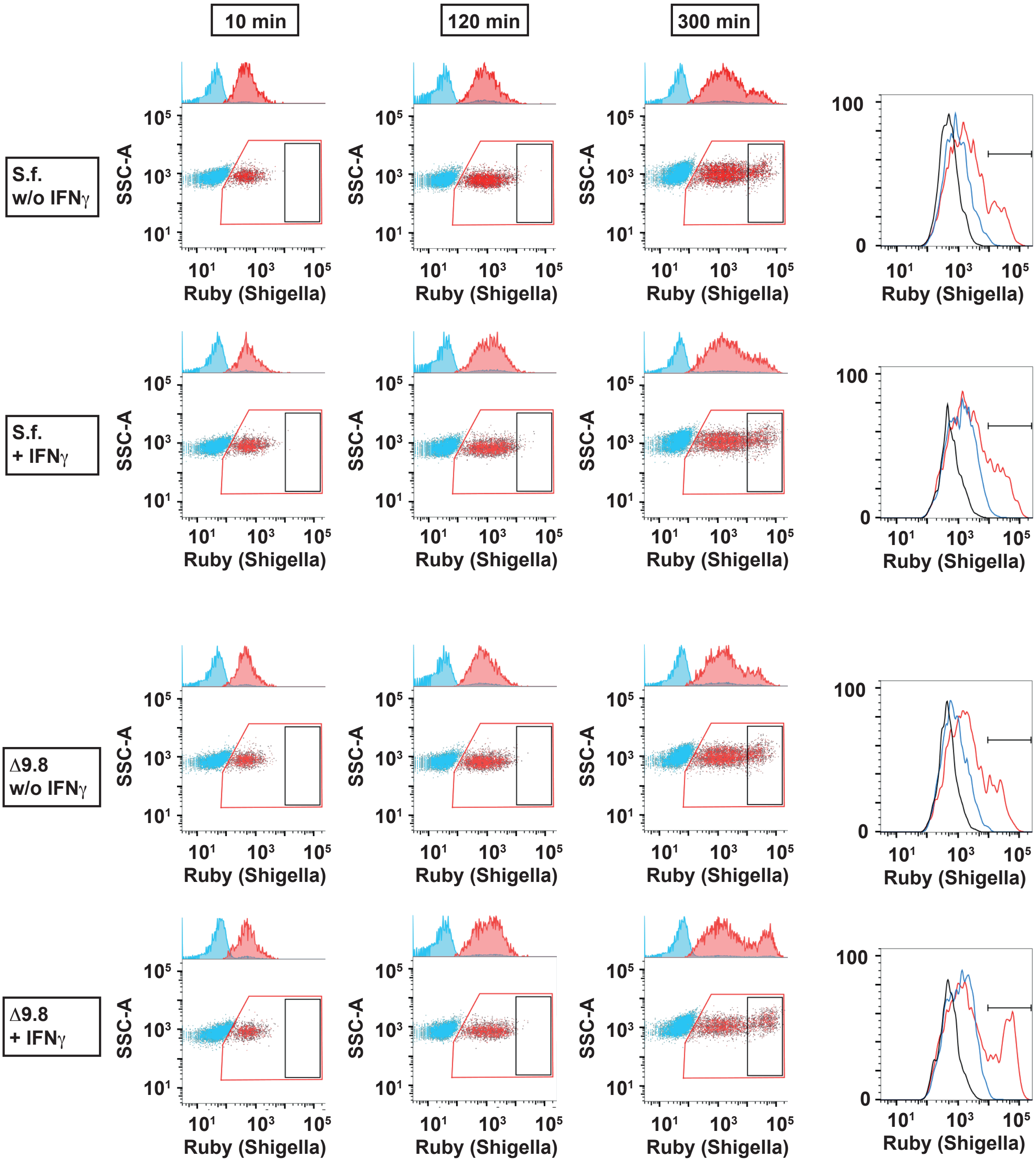

Oligonucleotides	Source	Identifier
Stealth RNAi™ siRNA Negative Control, Med GC	Thermo Fisher Scientific	Cat# 12935300
siSTAT1 #73 5’-GGAUUGAAAGCAUCCUAGAACUCAU	Thermo Fisher Scientific	Cat# HSS110273
siSTAT1 #74 5’-CCUGUCACAGCUGGAUGAUCAAUAU	Thermo Fisher Scientific	Cat# HSS110274
siSTAT1 #28 5’-GCGGAGACAGCAGAGCGACUGUAUU	Thermo Fisher Scientific	Cat# HSS186128
siGBP1 #21 5’-ACGGUGCAGUCUCACACUAAAGGAA	Thermo Fisher Scientific	Cat# HSS104021
siGBP1 #49 5’-GAGGCCAUUGAAGUCUUCAUCAGGA	Thermo Fisher Scientific	Cat# HSS178149
siGBP2 #23 5’-GGAGGAUGUGGCUGAUGCACUUCUA	Thermo Fisher Scientific	Cat# HSS104023
siGBP2 #25 5’-CCAAGAGGAAGUGCUUCGUCUUCGA	Thermo Fisher Scientific	Cat# HSS104025
siGBP3 #26 5’-GGGAGGCCACUGAAGUCUAUAUGAA	Thermo Fisher Scientific	Cat# HSS104026
siGBP3 #27 5’-GAACACACCUUAGUCCUGCUUGACA	Thermo Fisher Scientific	Cat# HSS104027
siGBP4 #00 5’-UCAGAGAGCAUGAAAGGCUGCUAAA	Thermo Fisher Scientific	Cat# HSS133000
siGBP4 #13 5’-CGGCCUACAAAUGACAAGCAAUAUU	Thermo Fisher Scientific	Cat# HSS174413

Table S2. siRNAs used for gene silencing. Refers to STAR Methods Key Resources Table.
