# Supplementary figures and images for: GBPs Inhibit Motility of Shigella flexneri but Are Targeted for Degradation by the Bacterial Ubiquitin Ligase IpaH9.8
Source: Cell Host Microbe. 2017 Oct 11;22(4):507–518.e5. doi: 10.1016/j.chom.2017.09.007 (PMC5644667; doi:10.1016/j.chom.2017.09.007)

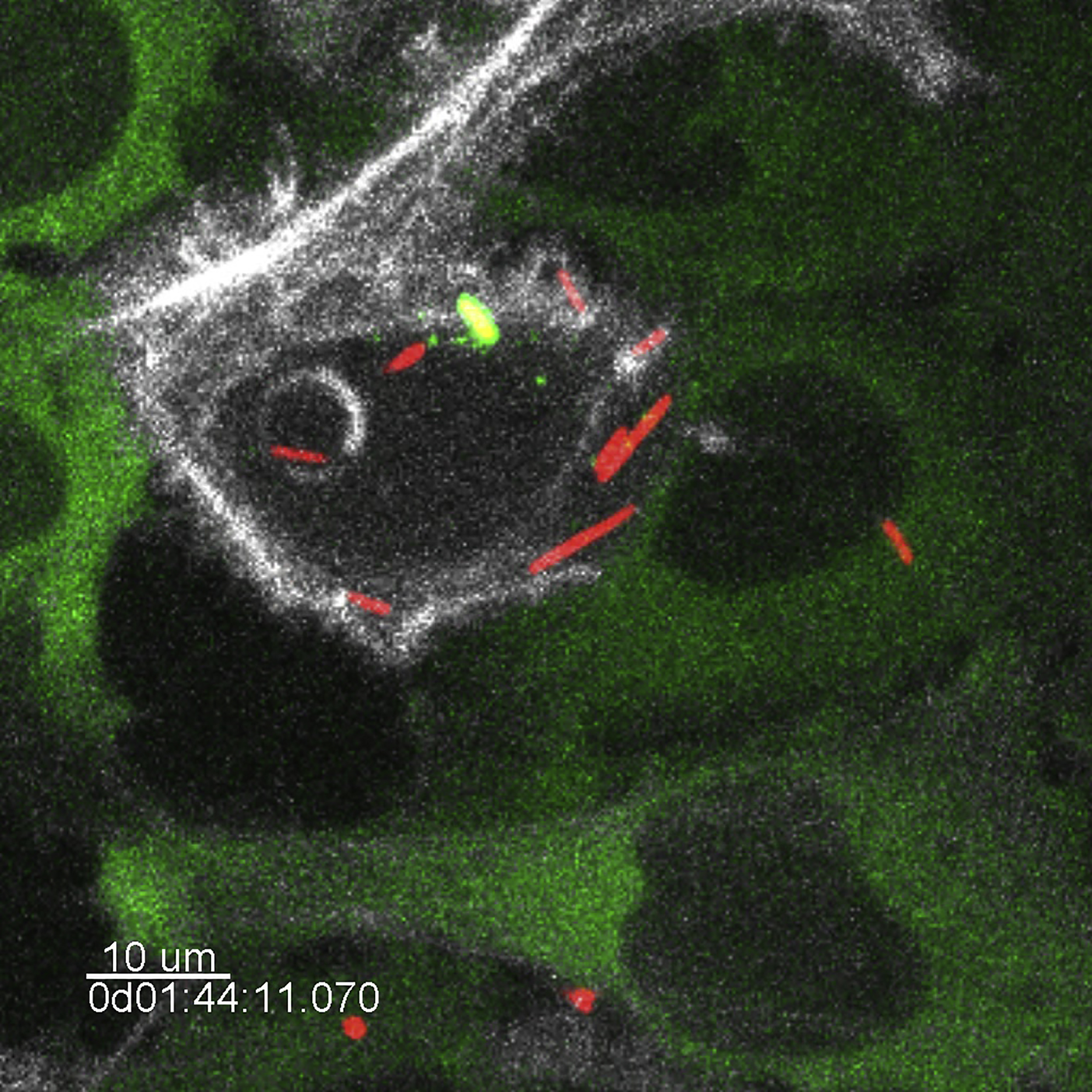

Supplement: Movie S1. Wild-Type S. flexneri Develop Actin Tails and Become Motile Once Their GBP1 Coat Has Been Degraded, Related to Figure 6 — Live imaging on a confocal spinning disk microscope of cells expressing GFP::GBP1 and Lifeact::CFP, infected with Ruby-expressing S. flexneri and imaged every 2 min. Scale bar 10 μm. [file mmc3.jpg]

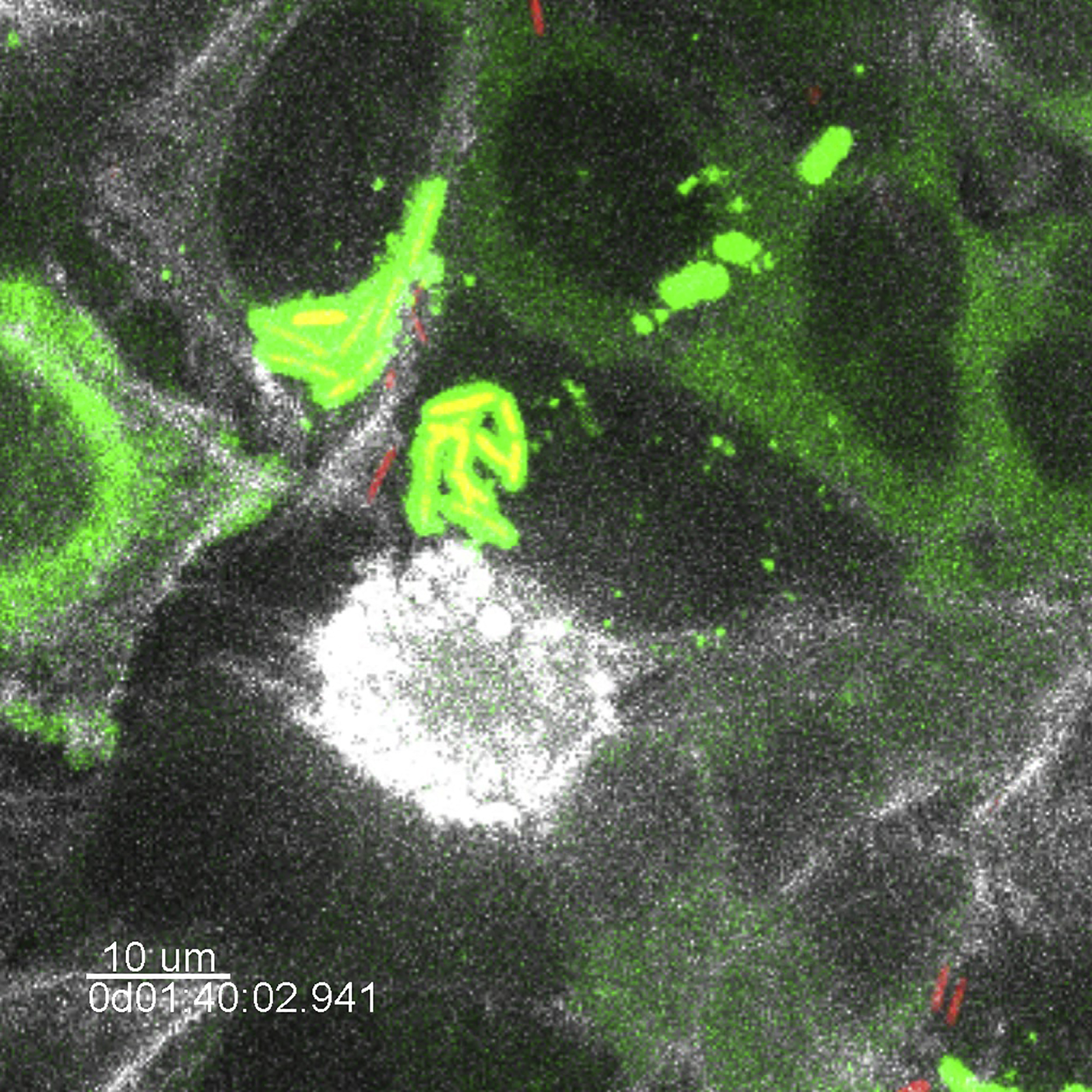

Supplement: Movie S2. S. flexneri ΔipaH9.8 Do Not Degrade Their GBP1 Coat and Remain Immotile, Related to Figure 6 — Live imaging on a confocal spinning disk microscope of cells expressing GFP::GBP1 and Lifeact::CFP, infected with Ruby-expressing S. flexneri ΔipaH9.8 and imaged every 2 min. Scale bar 10 μm. [file mmc4.jpg]
